# Research Progress on Quinone Compounds for the Treatment of Hepatocellular Carcinoma

**DOI:** 10.3390/biom15101400

**Published:** 2025-10-01

**Authors:** Yaowu Ye, Mengmeng Liu, Yukang Miao, Ke Pei, Zhe Lin, Songyan Liu, Xiaowei Huang, Yuchen Wang, Guangfu Lv

**Affiliations:** 1 School Pharmaceutical Sciences, Changchun University of Chinese Medicine, Changchun 130117, China; hxufnrhx@gmail.com (Y.Y.); thithanhd967@gmail.com (M.L.); maileduc12920@gmail.com (K.P.); linzhe@ccucm.edu.cn (Z.L.); huangxw@ccucm.edu.cn (X.H.); 2 Jilin Province Ginseng Science Research Institute, Changchun University of Chinese Medicine, Changchun 130117, China; buihoanghuy083@gmail.com; 3 Changchun Institute of Anticancer Drugs, Changchun 130033, China; aq888405@gmail.com

**Keywords:** hepatocellular carcinoma, quinone compounds, potential therapeutic strategies, medical application, bioactivity, pharmacological mechanism

## Abstract

Hepatocellular carcinoma (HCC) is the third most common cancer worldwide, widely prevalent across many countries, and poses a serious threat to human health. With changes in its epidemiology, the incidence of HCC is expected to continue rising. As a class of organic molecules widely distributed in nature, quinone compounds possess notable antioxidant, antibacterial, and antitumor properties. This article selects several quinone compounds that have shown notable research progress in recent years and artificially categorizes them into “plant-derived quinone compounds” and “non-plant-derived quinone compounds.” We then provide a detailed review of the research findings regarding HCC in vitro and in vivo experiments and clinical trials, including their potential toxic side effects. Additionally, based on the varying toxicity reduction of several selected plant-derived quinones when combined with doxorubicin, we further hypothesize that these plant-derived quinone compounds may also exert detoxifying effects on other non-plant-derived quinones discussed in this article. In summary, quinone compounds still hold significant research value and development potential in the fight against HCC. At the same time, we hope our review will provide valuable insights and inspiration for future research in this field.

## 1. Introduction

Hepatocellular carcinoma (HCC) is the third most common cancer worldwide, with an estimated 865,000 new cases diagnosed in 2022 and approximately 757,938 associated deaths [1,2]. As a significant threat to human health, HCC ranks among the top five leading causes of cancer-related mortality in 90 countries worldwide [3]. The epidemiology of HCC is currently undergoing significant shifts. While changes in susceptibility factors have contributed to a decline in hepatitis B and C as predominant etiological drivers, the rising prevalence of non-alcoholic fatty liver disease (NAFLD), also referred to as metabolic-associated fatty liver disease (MAFLD), is expected to drive a continued increase in HCC incidence over the next three decades [4].

Although the incidence of HCC remains concerning, significant advancements have been made in the clinical management of HCC over the past decade. Currently, treatment strategies for HCC primarily follow the Barcelona Clinic Liver Cancer (BCLC) staging system, which allocates different therapeutic approaches based on tumor staging. In principle, early-stage HCC patients are primarily treated with surgical resection, local ablation, or transplantation. For patients with intermediate-stage HCC, transarterial chemoembolization (TACE) is the preferred option, while advanced-stage patients are more likely to opt for systemic therapies. For those with compensated liver disease, surgical resection and systemic treatment are commonly employed to prevent liver function deterioration. Statistically, these treatment modalities have significantly improved the median survival time of various HCC patient groups [5].

Quinones are a class of organic compounds widely distributed in nature, characterized by their unique conjugated cyclic diketone structure, which imparts distinct physicochemical and biological properties. These compounds exhibit various bioactivities, including antioxidant, antibacterial, and antitumor effects. Due to their diverse functional potential, quinones have found promising applications in various fields such as industrial manufacturing, healthcare, and food technology. Consequently, research on quinone compounds has attracted growing interest in recent years [6,7]. However, despite numerous studies demonstrating the potential therapeutic efficacy of various quinone compounds against hepatocellular carcinoma (HCC), a comprehensive review summarizing their anti-HCC effects and mechanisms of action in HCC-related cellular models is still lacking.

This review categorizes representative quinone compounds into non-plant-derived and plant-derived quinones. We evaluate each category’s therapeutic advantages and limitations in treating hepatocellular carcinoma (HCC). We draw from evidence across three key domains: in vivo and in vitro studies, clinical research, and reported adverse effects. In addition, we examine the detoxifying properties of several plant-derived quinones in mitigating doxorubicin-induced toxicity. Finally, we provide a forward-looking perspective on the potential synergistic application of plant- and non-plant-derived quinones in HCC therapy.

## 2. Quinone Compounds

Quinonoid compounds constitute a broadly distributed class of organic molecules across various organisms, including plants, fungi, bacteria, and animals [8]. These compounds are secondary metabolites derived from the oxidative transformation of phenolic substances. Structurally, they are defined by a core framework featuring at least two carbonyl groups conjugated with adjacent double bonds, typically arranged within an aromatic or cyclic system. In higher plants, their biosynthesis involves multiple metabolic pathways. The structural diversity of these compounds is chiefly influenced by the degree of extension of their conjugated double-bond systems and the periodic arrangement of carbonyl groups. Accordingly, they can be categorized into four subclasses: benzoquinones, naphthoquinones, anthraquinones, and phenanthrenequinones. Existing literature data indicate that the distribution of quinonoid compounds in the plant kingdom exhibits significant family- and genus-specificity. These compounds are predominantly enriched in families such as Rubiaceae, Boehmeriaceae, and Polygonaceae, while secondary accumulation is observed in taxa including Liliaceae, Oleaceae, Meliaceae, Rosaceae, and Loranthaceae [9].

Regarding biological activity, quinonoid compounds exhibit significant antibacterial, antimalarial, and antitumor effects. Current studies suggest that these compounds may inhibit cancer progression and malignancy through multiple mechanisms, including inhibition of glycoproteins, DNA damage, cell cycle arrest induction, and apoptosis promotion [10,11]. Furthermore, quinonoid compounds may exert potent cytotoxicity, directly killing tumor cells. For example, Lee et al. isolated three phenanthrenequinone derivatives (calanquinone A, B, and C), among which calanquinone A demonstrated potent in vitro cytotoxicity against seven human cancer cell lines [12]. For instance, the quinonoid compounds Morindaparvin E and Morindaparvin F have demonstrated potent cytotoxic activity against various cancer cell lines, including HeLa cervical cancer cells and ovarian, renal, and breast cancer cells [13]. Although quinonoid compounds have been extensively investigated for their anticancer properties, the number of reported compounds with confirmed efficacy against primary hepatocellular carcinoma remains remarkably limited.

**Figure 1:**
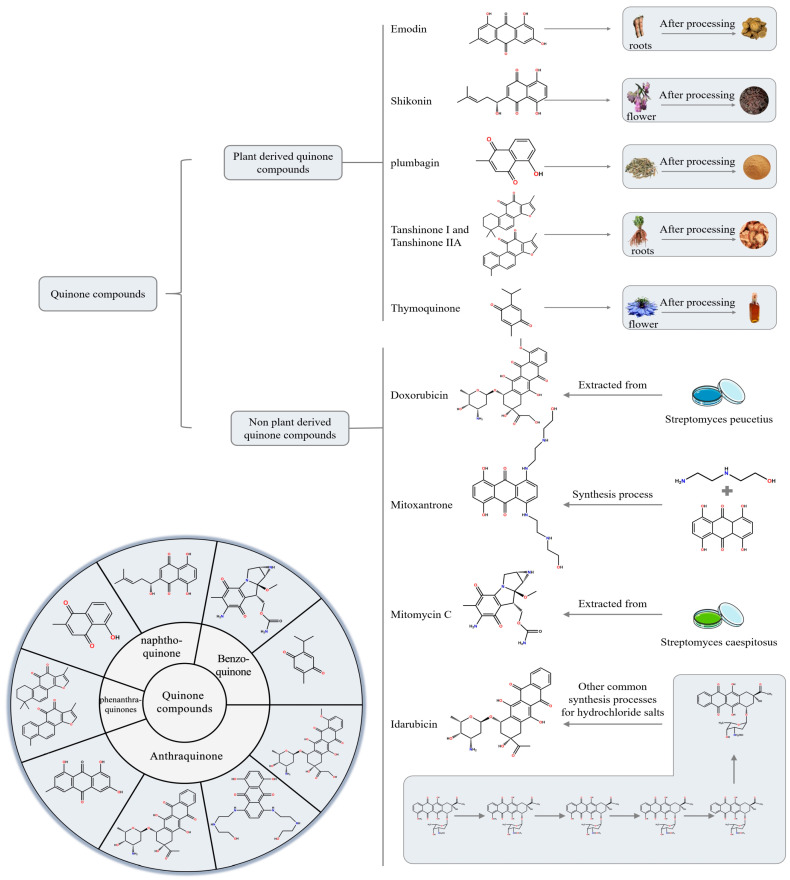
Classification of quinone compounds and their synthesis routes or source.

Based on a comprehensive search of databases including PubMed, Google Scholar, and ScienceDirect, we categorized nine predominantly reported quinonoid compounds with anti-HCC activity into plant-derived and non-plant-derived quinonoid compounds. We focused on highlighting the research progress of several key plant-derived quinonoids—emodin, shikonin, plumbagin (PLB), thymoquinone (TQ), tanshinone IIA (Tan IIA), and tanshinone I (Tan I)—and, among the non-plant-derived quinonoids, doxorubicin (DOX), mitoxantrone (MTX), idarubicin (IDA), and mitomycin (MMC). Figure 1 illustrates these quinonoid compounds’ sources, classifications, and chemical structures.

## 3. Research Progress of Plant-Derived Quinonoid Compounds in the Treatment of HCC

### 3.1. Experimental Studies of Plant-Derived Quinone Compounds in the Field of Anti-HCC

As one of the most lethal cancers worldwide, HCC remains only partially understood in terms of its biological and molecular mechanisms, despite extensive research efforts. The development of novel therapeutic agents for HCC must therefore be grounded in a deeper understanding of its underlying biology and molecular pathways. As the following section will discuss therapeutic strategies of quinone compounds against liver cancer, it will involve several key mechanisms, including apoptosis, oxidative stress, and epithelial–mesenchymal transition (EMT); to facilitate readers’ comprehension, we briefly summarize the major anti-HCC mechanisms of the quinone-based compounds discussed in this paper. Apoptosis, a form of programmed cell death, has long been a focal point in cancer research. Notably, the evasion of apoptosis is a key mechanism underlying drug resistance and disease progression in HCC. Thus, elucidating the molecular mechanisms responsible for dysregulated apoptosis in HCC cells is of critical importance [14]. Oxidative stress is a state of imbalance between pro-oxidant and antioxidant processes in the body, and it plays a distinct role in the development of HCC. During oxidative stress, large amounts of reactive oxygen species (ROS) are produced, acting as a double-edged sword. On the one hand, ROS can promote the proliferation and migration of tumor cells; on the other hand, excessive oxidative stress can lead to tumor cell death [15]. EMT is a multistep biological process that commonly occurs during embryonic development. In the progression of HCC, the harsh microenvironment within the tumor core induces EMT in hepatocytes, enabling them to acquire enhanced resistance to apoptosis and increased migratory capacity. Furthermore, EMT facilitates angiogenesis in HCC cells, allowing them to obtain oxygen and nutrients necessary for tumor growth. Therefore, the development of novel therapeutic agents should be based on a thorough understanding of the biological and molecular mechanisms underlying EMT [16]. Among the various cancer-related biological mechanisms, cuproptosis represents a relatively unique form of cell death. First defined in detail in 2022, cuproptosis has since been reported as a mechanism that can be exploited to induce cell death in lung and colorectal cancer cells. Given its distinct mode of action, the exploration of cuproptosis-related pathways holds great potential for future research and therapeutic development in the field of HCC [17].

Natural products have long served as valuable sources for developing novel therapeutics for various diseases, including cancer. Emodin, a naturally occurring anthraquinone derivative extracted from medicinal plants such as *Rheum* (rhubarb), *Polygonum cuspidatum*, and *Fallopia multiflora*, has demonstrated notable anti-hepatocellular carcinoma (HCC) activity. Qin, Hassan, et al. [18,19] demonstrated that emodin inhibits the proliferation of Hep-G2 cells by inducing cell cycle arrest at the S/G2-M phase and promoting apoptosis. In addition, emodin significantly suppresses these cells’ migratory and invasive capacities, improves the survival rate of HCC-bearing rats, and reduces the number of hepatic nodules. At the molecular level, emodin downregulates the elevated mRNA and protein expression of Protein Kinase C (PKC), Extracellular signal-regulated kinase 5 (ERK5), A Disintegrin And Metalloproteinase With Thrombospondin 4 (ADAMTS4), Matrix Metalloproteinase 3 (MMP3), and Vascular Endothelial Growth Factor (VEGF) in the livers of affected rats. Beyond its intrinsic anti-HCC activity, emodin exhibits promising potential with conventional chemotherapeutic agents for treating HCC. A study conducted by Kim et al. [20] demonstrated that emodin inhibits the transcriptional activity of sterol regulatory element-binding protein 2 (SREBP-2), thereby suppressing cholesterol biosynthesis and downstream signaling of the oncogenic protein kinase B (AKT). Furthermore, when combined with sorafenib, emodin synergistically enhances sorafenib-induced G1 phase cell cycle arrest and apoptosis. In vivo, emodin exhibits superior tumor growth inhibition in animal models xenografted with Hep-G2 or SK-HEP-1 cells. Similarly, cisplatin is a cytotoxic anticancer drug with broad-spectrum efficacy. It exerts its effects by inhibiting DNA replication in cancer cells and disrupting structural components of their cell membranes [21,22]. However, its clinical application is limited due to the development of drug resistance and inherent toxicity [23]. YANG et al. [24] investigated the combined application of cisplatin and emodin and found that the combination exerted significantly stronger inhibitory effects on cell proliferation than either agent alone. Moreover, the co-treatment more effectively suppressed cell migration in wound healing assays, exhibiting both time- and dose-dependent responses. This enhanced antitumor efficacy is likely attributed to emodin’s ability to inhibit epithelial–mesenchymal transition (EMT), thereby increasing the sensitivity of Hep-G2 cells to cisplatin. In summary, emodin inhibits HCC progression by modulating key signaling pathways, such as PKC/ERK5/MMP3. Combined with agents like sorafenib or cisplatin, it synergistically enhances cell cycle arrest, apoptosis, and chemosensitivity. Nevertheless, further mechanistic studies and comprehensive safety evaluations must support its clinical translation.

Shikonin is a naturally occurring red-colored quinonoid compound extracted from the roots of Lithospermum erythrorhizon (commonly known as purple gromwell). It exhibits a broad spectrum of pharmacological activities, including anti-inflammatory, antioxidant, and anticancer effects [25]. Mitochondria play a critical role in regulating numerous aspects of cellular function, including energy metabolism, apoptosis, and redox balance. Mitochondrial dysfunction has been shown to significantly affect cell growth and proliferation, contributing to the pathogenesis of HCC [26]. Shikonin has been reported to induce apoptosis in HCC cells by increasing reactive oxygen species (ROS) production by inhibiting pyruvate kinase M2 (PKM2) activity. However, the sensitivity to shikonin varies among different HCC cell lines, suggesting cell line-specific mechanisms of action or resistance. Therefore, Yang et al. [27] evaluated the effects of shikonin on oxidative stress and metabolic pathways in sensitive and resistant HCC cell lines. They sought to identify the mechanisms underlying the differences in sensitivity. The study results indicate that Hep-G2 cells are more sensitive to shikonin than HCCLM3 cells. Shikonin induces nuclear expression of PKM2 and HIF1α, activating glycolysis in HCCLM3 cells but not in Hep-G2 cells, while downregulating PGC1α-mediated mitochondrial biogenesis to enhance cell survival. This may be the primary reason HCCLM3 cells are less sensitive to shikonin than Hep-G2 cells. In summary, shikonin disrupts the energy metabolic homeostasis of HCC cells through multiple molecular targets, thereby providing a critical theoretical foundation for developing metabolism-oriented precision therapies for HCC.

Plumbagin (PLB) is a naturally occurring naphthoquinone isolated from the plant Plumbago zeylanica L. It has been demonstrated to possess anticancer activity against hepatocellular carcinoma and various other types of cancer [28]. The findings of Liu et al. [28] demonstrate that PLB exerts inhibitory effects on the viability of both Huh-7 and Hep-G2 HCC cell lines, concurrently reducing their colony formation capacity. Moreover, PLB induces oxidative stress and DNA damage in HCC cells by elevating intracellular ROS levels and activating cell cycle checkpoints. Building upon the observation that PLB inhibits the proliferation of HCC cells, though the underlying mechanisms remained unclear, Yao et al. [29] conducted a series of in vitro and in vivo experiments to elucidate the molecular basis of this effect. Their findings revealed that PLB suppresses the deubiquitinase activity of USP31, thereby promoting the ubiquitination and subsequent destabilization of glutathione peroxidase 4 (GPX4). The reduced stability of GPX4 leads to a marked accumulation of reactive oxygen species (ROS), ultimately inducing apoptosis in HCC cells. In a separate study, Du et al. [30] investigated the impact of PLB on EMT in HCC cells. Their results revealed that PLB significantly upregulates epithelial markers while downregulating mesenchymal markers. Western blot analysis of EMT-related proteins further confirmed the potent inhibitory effect of PLB on the EMT process in HCC cells. Additionally, in vivo experiments demonstrated that PLB markedly reduces the expression of Snail, vimentin, and N-cadherin. Histopathological examination showed that the number of hepatic tumor foci and pulmonary metastatic lesions in the PLB-treated group was substantially lower than in the control group, providing more direct evidence of PLB’s antitumor efficacy against HCC. Cuproptosis is a novel mode of cell death first comprehensively characterized in 2022. The accumulation of copper within mitochondria facilitates the aggregation of lipoylated dihydrolipoamide S-acetyltransferase (DLAT), triggering this distinct form of cell death. Against the backdrop of cuproptosis research, Wang et al. [17] sought to elucidate the potential involvement of this novel cell death pathway in the antitumor effects of PLB on HCC. Their findings demonstrated that PLB treatment leads to the transcriptional downregulation of ATP7B, a key regulator of intracellular copper homeostasis. This downregulation results in copper accumulation within HCC cells, ultimately triggering cuproptosis. Further mechanistic investigations revealed that PLB induces the downregulation of DNA methyltransferase 1 (DNMT1), thereby promoting the transcription of miR-302a-3p. The upregulated miR-302a-3p subsequently binds to the 3′untranslated region (3′UTR) of ATP7B mRNA, leading to its silencing. This molecular cascade culminates in copper overload and the induction of cuproptosis in HCC cells. In the context of combination therapy with other anti-HCC agents, PLB has been shown to act synergistically with non-plant-derived quinone compounds, such as DOX, to suppress HCC progression. Cao et al. [31] initially confirmed the synergistic cytotoxic effects of DOX and PLB when co-administered in HCC chemotherapy. They subsequently developed a targeted nanoformulation incorporating DOX, PLB, and PLGA-PEG-AEAA. They evaluated its efficacy using both in vitro cell models and a subcutaneous DOX-resistant HCC mouse model. The results demonstrated that, compared to monotherapy, the combination of DOX and PLB synergistically induced apoptosis in DOX-resistant Hepa1-6 cells (Hepa1-6-R cells) and significantly inhibited their migratory capacity. This enhanced efficacy may be attributed to PLB-mediated suppression of aberrant STAT3 activation. Furthermore, compared to non-targeted formulations and the combination of free DOX and PLB, the targeted formulation induced greater apoptosis and more effectively reduced cell migration in Hepa1-6-R cells. In subsequent in vivo experiments, the targeted formulation significantly reduced tumor weight and STAT3 activity within tumor tissues. This study established a mechanistic and therapeutic link between plant-derived and non-plant-derived quinones in the combinatorial treatment of HCC, thereby advancing research on quinone-based combination strategies for liver cancer therapy. Dan Shen is one of China’s most popular traditional herbs and contains various natural quinone compounds. Tanshinone I (Tan I) is one of the well-known major active constituents of Salvia miltiorrhiza. In recent years, it has attracted considerable attention in the research community due to emerging studies. It has been shown to possess potent activities such as antioxidative stress effects, autophagy and apoptosis regulation, and inflammation inhibition [32]. Tanshinone IIA (Tan IIA), a quinonoid bioactive compound from Salvia miltiorrhiza (Danshen), has attracted significant scholarly attention due to its unique structure and potent pharmacological effects. Extensive research has revealed its wide-ranging therapeutic properties, including anti-inflammatory, antioxidant, antiarrhythmic, cardioprotective, and antitumor activities. In recent years, notable progress has been made in understanding its mechanisms and efficacy, particularly in antitumor applications [33]. In studies investigating the therapeutic effects of Tan I on HCC, this compound has been shown to induce G0/G1 phase cell cycle arrest and subsequent apoptosis in liver cancer cells by downregulating cyclin D1 and upregulating p21 expression. Additionally, Tan I triggers apoptosis in Hep-G2 and Huh-7 cells by promoting endoplasmic reticulum (ER) stress through generating reactive oxygen species (ROS) and inhibiting p53-mediated autophagy. Furthermore, another study demonstrated that Tan I impairs genomic stability in HCC cells by suppressing non-homologous end joining (NHEJ) and homologous recombination (HR) DNA repair pathways in a dose-dependent manner [34,35]. Epirubicin (EADM) is a commonly used chemotherapeutic agent for treating hepatocellular carcinoma (HCC); however, like many other anti-HCC drugs, it often leads to the gradual development of drug resistance in cancer cells. The accumulation of hypoxia-inducible factor-1 alpha (HIF-1α) has been identified as a key contributor to EADM resistance in HCC. Several studies have reported that certain tanshinones may modulate HIF-1α expression by targeting the PI3K/AKT signaling pathway [36]. Building upon this research background, Zhao et al. demonstrated that the combined application of Tan I and epirubicin (EADM) enhances the cytotoxicity and growth inhibitory effects of EADM against HCC cells by targeting the PI3K/AKT/HIF-1α signaling pathway. Notably, the combination synergistically reverses HIF-1α-mediated drug resistance, partially overcoming EADM resistance in hepatocellular carcinoma. Regarding safety, in vivo studies in mouse models confirmed that the combination therapy is both safe and effective, and it also mitigates the body weight loss typically induced by EADM treatment [37]. In recent years, PD-1 inhibitors have gained widespread popularity as a treatment option for various tumors. However, their efficacy in treating HCC has been limited [38]. This phenomenon may be attributed to the limited infiltration capacity of tumor-infiltrating lymphocytes (TILs) within the tumor microenvironment, potentially due to abnormal tumor vasculature. Although previous studies have shown that Tan IIA can enhance vascular integrity and promote vascular normalization, the precise molecular mechanisms underlying these effects remain poorly understood [39]. Mao et al. [40] reported that tanshinone IIA (Tan IIA) enhances vascular integrity in situ by promoting the expression of tight junction proteins—ZO-1, Occludin, and Claudin-5—through inhibition of ELTD1 expression, while concurrently suppressing JAK1 and JAK2 signaling pathways. These effects contribute to the remodeling of the immunosuppressive tumor microenvironment and the inhibition of tumor growth. Furthermore, combining Tan IIA with PD-1 inhibitors resulted in significantly greater tumor suppression than PD-1 inhibitor monotherapy in animal models. However, relatively few studies have explored the synergistic effects of Tan IIA in combination with conventional chemotherapeutic agents. Although these studies remain preliminary, emerging evidence suggests that combining Tan IIA with sorafenib or its derivative SC-1 significantly suppresses cell migration, invasion, and sorafenib resistance in HCC cells [41]. The observed reduction in drug resistance is likely attributable to the combination’s potent inhibition of STAT3 signaling. Furthermore, this combined treatment increases the sub-G1 cell population in Huh-7 and Hep-G2 cells, accompanied by pronounced activation of caspases, indicating enhanced apoptosis.

**Figure 2:**
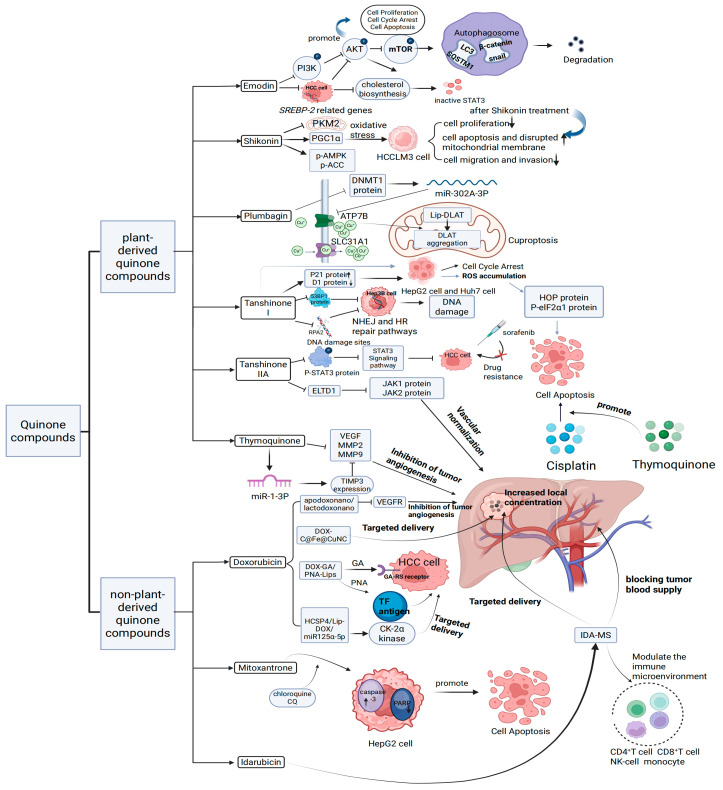
Mechanism of action of plant-derived quinones and non-plant-derived quinones on HCC.

TQ is a plant-derived natural quinone compound extracted from Nigella sativa [42]. Due to its antioxidant, hepatoprotective, anticancer, and anti-inflammatory properties, there is growing interest in its pharmacological mechanisms and therapeutic effects [43]. Studies by Ibrahim, Tadros, and Farghaly [44,45,46] suggest that TQ may inhibit angiogenesis in HCC by regulating miR-1-3p. Additionally, data from related animal models indicate that TQ effectively prevents changes in liver enzymes and serum proteins while reducing the expression levels of HCC markers such as AFP, AFP-L3, and GPC3. Furthermore, TQ significantly decreases BCL-2 expression and upregulates the BAX/BCL-2 ratio and *CASP3* expression levels. Regarding combination therapies involving common anti-HCC chemotherapeutic agents, TQ combined with cisplatin has significantly improved liver pathology. TQ enhances the efficacy of cisplatin by mitigating oxidative stress and downregulating the endoplasmic reticulum stress marker GRP78, thereby substantially reducing cisplatin-induced oxidative damage. Moreover, TQ inhibits angiogenesis in HCC and slows disease progression by modulating multiple signaling pathways and molecular targets. Significantly, TQ not only potentiates the therapeutic effects of cisplatin but also attenuates some of the drug’s adverse side effects. The relevant mechanisms underlying the anti-HCC effects of these plant-derived quinone compounds are summarized in Figure 2. Additionally, the sources of these compounds and the outcomes of corresponding animal and cellular studies are briefly detailed in Table 1.

### 3.2. Clinical Studies on Plant-Derived Quinonoid Compounds

Investigations into the anti-HCC effects of the aforementioned plant-derived quinonoid compounds have primarily been confined to in vitro cellular assays and animal models, resulting in relatively limited clinical data on their efficacy as individual agents. Nevertheless, the source plants of these quinones are frequently incorporated into compound formulations currently used in clinical management of HCC, offering valuable insights and reference points for future research and therapeutic innovation. For example, in formulations such as the Da Huang Zhe Chong Formula and rhubarb-based sodium tablets studied by Hou, Tian, Deng, et al. [47,48,49], the emodin present in rhubarb may contribute to therapeutic effects in patients with HCC. Notably, post-embolization syndrome following TACE was significantly reduced in HCC patients treated with the Da Huang Zhe Chong Formula. Additionally, patients receiving interventional therapy for liver cancer exhibited marked improvements in liver function and enhanced immune responses. Furthermore, treatment with rhubarb-based sodium tablets effectively improved serum biomarkers, including ALT, TBIL, and AFP, while significantly reducing complications such as upper gastrointestinal bleeding.

Danshen injection is a traditional Chinese medicinal preparation primarily derived from Salvia miltiorrhiza, the primary source of Tan I and Tan IIA. Clinical studies conducted by Zhu, Zhang, et al. [50,51] have demonstrated that Danshen injection may enhance immune function in patients with intermediate to advanced HCC by increasing levels of CD4+ T cells, CD3+ T cells, the CD4+/CD8+ T cell ratio, and serum immunoglobulins IgM, IgG, and IgA. Furthermore, Danshen injection has been reported to improve clinical symptoms and signs and to prolong patient survival (Table 2).

### 3.3. Adverse Reactions of Plant-Derived Quinonoid Compounds

Although plant-derived quinone compounds have shown preliminary potential to inhibit HCC via multiple pathways and targets, they are not without specific toxicities. The toxic effects of these compounds may pose significant challenges to their advancement into clinical trials. Among them, emodin—a natural plant-derived quinone with anticancer properties—has been reported to exhibit fewer side effects compared to conventional chemotherapeutic agents [52]. The primary toxicities associated with emodin include reproductive toxicity, genotoxicity, and mutagenicity. Oshida et al. found that emodin can impair spermatogenic function in mice and interfere with the expression of genes related to spermatogenesis. Drinking an emodin solution at a concentration of 20–40 μM can reduce the maturation rate of mouse oocytes and the in vitro fertilization rate, thereby damaging early embryonic development. When emodin concentration exceeds 0.93 μM, the compound adversely affects the embryo survival rate and hatching rate of zebrafish [53,54,55]. The toxicity of shikonin primarily manifests as skin toxicity, cytotoxicity, hepatotoxicity, and metabolic toxicity. Cheng et al. [56] and Figat et al. [57] reported that shikonin acts as an inhibitor of uridine 5′-diphosphate glucuronosyltransferase (UGT), contributing to hepatotoxicity in both humans and rats. Additionally, shikonin exhibits potent cytotoxicity against the conventional V79 cell line. Tang et al. [58] further demonstrated that shikonin may inhibit multiple cytochrome P450 enzymes in mammals. Acting as a mixed and competitive inhibitor of CYP1A2, CYP2C9, CYP2B6, CYP2D6, CYP3A4, and CYP2E1, shikonin has the potential to interfere with drug metabolism and cause drug–drug and food–drug interaction toxicities. The toxicity profile of PLB is primarily characterized by reproductive toxicity in female animals and, to a lesser extent, hepatotoxicity. In addition, a limited number of studies have reported that PLB may also exhibit considerable genotoxicity and antigenic toxicity [59]. Kumar et al. [60] administered three crude extracts of PLB to Wistar rats, using petroleum ether, acetone, and 70% ethanol as extraction solvents. The experimental results showed that all PLB extracts induced hepatic and renal congestion in the animals, accompanied by elevated levels of biochemical markers such as AST and urea. Notably, since the petroleum ether extract contained the highest concentration of PLB, rats receiving this extract at equivalent maximum doses exhibited more rapid mortality. Lim et al. [61] focused specifically on the reproductive toxicity of PLB. To assess their developmental toxicity, they investigated four 1,4-naphthoquinone derivatives, including PLB, by exposing both wild-type and transgenic zebrafish embryos. The study found that PLB treatment significantly increased embryo lethality and reduced hatching rates. Notably, when zebrafish embryos were exposed to 0.13 µM PLB at 4 h post-fertilization, severe impairment of brain differentiation and the manifestation of pronounced cyclopia were observed. Embryos exhibiting cyclopia ultimately failed to survive. The toxicity of TQ primarily manifests as genotoxicity and teratogenicity. Abukhader et al. [62] demonstrated that TQ administered at doses of 35 mg/kg and 50 mg/kg induced significant adverse effects on embryonic development during pregnancy in rats. Toxicological data and side effect profiles for Tan I, both in vivo and in vitro, remain insufficient and require further investigation [63]. However, Huang et al. [64] reported that all lipophilic tanshinones cause various developmental defects in zebrafish. Specifically, Tan IIA has been associated with teratogenic effects and potential cardiotoxicity. Wang et al. [65] observed that zebrafish embryos exposed to different concentrations of Tan IIA for four days exhibited pronounced pericardial edema, spinal curvature, and tail loss. Tan IIA showed evidence of cardiotoxicity at higher concentrations in zebrafish embryos. A summary of the sources, toxicities, and adverse effects of these plant-derived quinones is provided in Table 3.

## 4. Research Progress of Non-Plant-Derived Quinone Compounds in the Field of Anti-HCC

### 4.1. Experimental Studies of Non-Plant-Derived Quinone Compounds in the Field of Anti-HCC

Anthracyclines represent a class of aromatic type II polyketide compounds characterized by a linear tetracyclic 7,8,9,10-tetrahydro-5,12-naphthacenedione core. This scaffold features a polyhydroxylated anthraquinone structure fused with a fourth saturated substituted ring, forming the aglycone moiety typically conjugated to one or more sugar residues. The history of anthracyclines in cancer therapy dates back to the 1960s with the discovery of daunomycin, whose remarkable efficacy against lymphoma significantly propelled research into this class of molecules. Since then, continuous structural optimization and biosynthetic engineering—particularly through modified bacterial strains—have produced hundreds of anthracycline analogs. Beyond the three clinically relevant agents discussed in this paper—DOX and IDA, commonly used in HCC therapy—numerous other anthracyclines have been approved for clinical application. For instance, amrubicin (Amr) is employed in the treatment of lung cancer, while epirubicin is widely used in adjuvant therapy for hormone receptor-positive breast cancer. However, a major limitation of this class, particularly in the case of DOX, is its pronounced cardiotoxicity. Therefore, a central focus of current research is the development of strategies to mitigate this adverse effect while simultaneously enhancing the therapeutic efficacy of anthracyclines [66].

Doxorubicin is a cytotoxic anthracycline antibiotic isolated from Streptomyces peucetius cultures. Its anticancer activity primarily arises from intercalation into DNA nucleotide bases. Specifically, DOX disrupts the DNA replication process by stabilizing the topoisomerase II-DNA cleavage complex, thereby preventing the re-ligation of the DNA double helix and effectively terminating DNA replication [67]. Although its severe side effects limit the clinical application of DOX, recent advances in nanocarrier technologies may help mitigate these issues while enhancing its therapeutic efficacy. Bhaskar, Saddik, and colleagues [68,69] independently developed deferroprotein- and nano-lactoferrin-loaded DOX formulations and a DOX-loaded C@Fe@Cu nanocomposite. Their studies demonstrated that both types of protein-based DOX nanoparticles effectively treated HCC in rat models. Notably, the nanoprotein-treated groups exhibited significantly higher hepatic DOX concentrations than those receiving free DOX. Furthermore, these groups showed greater upregulation of p53 and p21 expression, suggesting enhanced apoptotic activity relative to DOX alone. In parallel, the C@Fe@Cu nanocomposite formulation markedly reduced the IC50 of DOX, indicating increased cytotoxic potency against liver cancer cells. Cells treated with the DOX-C@Fe@Cu nanocomposite also exhibited a higher proportion of late apoptotic cells than those treated with an equivalent dose of free DOX. These findings suggest that nanocarrier systems based on nanoproteins or metal–organic composites may offer significant advantages in augmenting the anti-HCC efficacy of DOX, presenting a promising new strategy for liver cancer therapy. Similarly, liposomes—widely regarded as non-toxic, biocompatible, and non-immunogenic drug carriers—also hold considerable potential [70,71]. Liposomes are widely utilized to encapsulate lipophilic and hydrophilic drugs, enhancing their pharmacokinetic and pharmacodynamic profiles to improve therapeutic efficacy and reduce systemic toxicity [72]. Li, Zhao, and colleagues [72,73] respectively developed DOX-loaded liposomal formulations modified with glycyrrhetinic acid (GA) and peanut agglutinin (PNA) as targeting ligands (DOX-GA/PNA-Lips), as well as an HCC-targeted liposomal delivery system designed to overcome multidrug resistance (MDR), termed HCSP4/Lipo-DOX/miR125a-5p. Their studies showed that DOX-GA/PNA-Lips significantly enhanced targeting efficiency toward the MUC1-positive HCC cell line SMMC-7721. In vivo, mice treated with the liposomal DOX formulation exhibited reduced tumor volume and weight and less body weight loss compared to those receiving free DOX. Additionally, the HCSP4/Lipo-DOX/miR125a-5p formulation effectively inhibited MDR development by downregulating the expression of MDR-associated genes. These findings highlight the potential of ligand-modified and gene-regulatory liposomal systems in enhancing DOX delivery and overcoming resistance mechanisms in HCC therapy. After treatment with HCSP4/Lipo-DOX/miR125a-5p, the expression levels of *ABCB1*, *ABCC5*, *ATP1B1*, and *EZH2* were significantly reduced in Hep-G2 and Hep-G2/ADR cells. In summary, this study provides a liposome-based DOX delivery strategy that enhances the targeting efficiency of HCC treatment and effectively inhibits the development of MDR, offering new ideas and experimental evidence for HCC therapy.

Due to the severe cardiotoxicity associated with DOX, significant research efforts have focused on mitigating its cardiac side effects through various strategies. They also pursue the development of novel anticancer agents with reduced cardiotoxic profiles. In the 1980s, MTX was introduced as a synthetic analogue of DOX [74]. As a member of the anthracycline class, MTX is widely used to treat malignancies such as lymphoma and bone marrow cancers. However, its clinical efficacy in HCC, particularly in patients with advanced-stage disease, has been limited [75]. Autophagy, an evolutionarily conserved cellular process, enables the degradation and recycling of intracellular components in response to stress and environmental stimuli [76]. This mechanism may contribute to the survival of HCC cells during MTX treatment by mitigating drug-induced cytotoxic stress [77]. Using HCC cells as a model, Xie et al. [77] observed a significant increase in LC3-II levels and a concomitant decrease in p62 levels following MTX treatment, indicating enhanced autophagic activity. In the Hep-G2 group treated with a low dose of MTX, an increase in Beclin-1 was observed, whereas in the high-dose MTX-treated Hep-G2 group, Beclin-1 levels slightly decreased. Additionally, in MTX-treated Hep-G2 cells, phosphorylation of p70S6K at Thr389 and total protein levels were significantly reduced. They then used chloroquine (CQ) to inhibit autophagy in MTX-treated Hep-G2 cells and measured apoptosis. Compared to the MTX-only group, the combined treatment group (MTX + CQ) showed a significantly higher cleavage rate, indicating that CQ enhanced MTX-induced apoptosis in Hep-G2 cells by inhibiting autophagy.

In the ongoing effort to develop anthracycline-based antitumor agents with reduced cardiotoxicity, IDA was introduced in 1976 [78]. Structurally, IDA differs from daunorubicin by substituting a hydrogen atom for the C4 methoxy group on the aglycone D-ring. This seemingly minor modification significantly increases the drug’s lipophilicity, leading to substantially higher plasma concentrations of its metabolites than daunorubicin [79]. Due to its improved lipophilicity and extended biological half-life, IDA is frequently employed in TACE to treat HCC. However, conventional TACE formulations often suffer from emulsion instability, which leads to rapid phase separation and inadequate local drug accumulation within the tumor site, thereby limiting therapeutic efficacy. To overcome the above limitations, Zheng et al. [80] developed a biodegradable microsphere loaded with idarubicin (IDA-MS). They then used IDA-MS for TACE treatment in VX2 rabbit and C57BL/6 mouse models. In the rabbit VX2 HCC model, TACE with IDA-MS significantly inhibited tumor growth and blocked tumor blood vessels more effectively. In the mouse HCC model, IDA-MS markedly reduced tumor volume and weight and improved the pathological condition of tumor tissues. This study indicates that a biodegradable microsphere-based IDA delivery system has potential advantages in TACE treatment of HCC, providing new experimental evidence for optimizing chemotherapy embolization strategies for HCC. The dosing form of the above non-plant-derived quinones and the associated mechanisms of anti-HCC action can be seen in Figure 2. The source, mode of administration, or form of the above non-plant-derived quinones and the corresponding efficacy are briefly documented in Table 4.

### 4.2. Clinical Studies of Non-Plant-Derived Quinone Compounds in the Field of Anti-HCC

DOX, a widely used anthracycline chemotherapeutic agent, exhibits potent antitumor activity against various malignancies. However, its clinical utility is severely limited by its significant systemic toxicity. In a study by Lai et al. [81], 60 patients with inoperable HCC were randomly assigned to receive DOX every three weeks, while 46 comparable patients received no anticancer therapy. Although the DOX-treated group demonstrated a higher median survival compared to the untreated group, approximately 25% of patients experienced fatal complications, underscoring the drug’s severe toxicity. To mitigate these adverse effects, researchers have explored liposomal formulations of DOX in treating HCC. Valle, Tak, and colleagues [82,83] evaluated two such approaches: pegylated liposomal doxorubicin (PLD) and lyso-thermosensitive liposomal doxorubicin (LTLD) in combination with radiofrequency ablation (RFA). Patients treated with PLD exhibited improved tolerance to several of DOX’s toxic side effects. Although the combination of RFA and LTLD did not show a significant safety advantage over RFA alone, its therapeutic efficacy was notably enhanced—particularly when RFA was applied to isolated lesions for 45 min or longer.

MTX has been used in the clinical treatment of HCC for the last century. Shepherd, Farres et al. [84,85] conducted hepatic arterial infusion of MTX and minimally invasive intratumoral injection of MTX in patients with HCC. Under the hepatic arterial infusion treatment, MTX demonstrated potent activity against primary HCC; However, the drug appeared to exhibit toxic effects similar to those of epirubicin. Meanwhile, in malignant hepatic lesions with no other therapeutic options, minimally invasive intratumoral injection of MTX promoted tumor necrosis and showed a high safety profile. With the rapid advancement of nanocarrier technology, clinical research on MTX treatment for HCC has also achieved breakthroughs. Zhou et al. [86] compared the therapeutic effects of Mitoxantrone (Dihydroxyanthracenedione, DHAD) delivered via polybutylcyanoacrylate nanoparticles (DHAD-PBCA-NPs) to those of free DHAD in patients with HCC. The DHAD-PBCA-NP group demonstrated improved clinical outcomes, including greater disease stabilization, reduced disease progression, and a lower incidence of leukopenia compared to the group receiving DHAD alone. However, the incidence of anemia was lower in the DHAD-treated group than in the DHAD-PBCA-NP group. Mitomycin C (MMC), a cytotoxic antibiotic originally isolated from Streptomyces caespitosus, has shown broad-spectrum antitumor activity and has long been investigated in the clinical management of HCC [87]. Clinical studies by Cheirsilpa et al. [88] revealed that high-dose MMC exhibited selective antitumor efficacy against HCC with a more favorable toxicity profile than doxorubicin. Idarubicin (IDA), a second-generation anthracycline, has also demonstrated superior cytotoxicity against HCC cells in preclinical in vitro studies [89]. Growing clinical evidence further supports the potential of IDA to replace DOX in transarterial chemoembolization (TACE) regimens for HCC. Boulin et al. [90] administered microspheres loaded with varying doses of IDA to patients via TACE. They observed that the maximum tolerated dose of IDA loaded on the drug-eluting beads was 10 mg per TACE session. This clinical study’s median overall survival was significantly higher than conventional TACE treatment. Furthermore, their findings demonstrated that IDA-loaded drug-eluting beads were more effective in treating HCC than intravenous administration of IDA. Teyssier et al. [91] retrospectively reviewed the medical records and imaging studies of HCC patients who consecutively underwent Doxorubicin-TACE (Dox-TACE) or Idarubicin-TACE (Ida-TACE) treatments at their hospital from 2012 to 2014. Comparing the objective response rates, complete response rates, and safety profiles of the two TACE treatments, the results indicated that Ida-TACE has promising potential as a superior alternative to Dox-TACE for treating intermediate-stage HCC.

In summary, although the aforementioned non-plant-derived quinones have demonstrated vigorous anti-HCC activity and have been combined with other antitumor therapies in subsequent studies to maximize their clinical effectiveness, their side effects, toxicity, and inability to achieve a cure for HCC remain significant obstacles to the future development of these drugs. The clinical efficacy of the above non-plant-derived quinones is briefly documented in Table 5.

### 4.3. Adverse Reactions and Toxicity of Non-Plant-Derived Quinone Compounds

In the early years of doxorubicin (DOX) clinical use, its most common side effects were identified as acute nausea and vomiting, stomatitis, alopecia, and gastrointestinal disturbances. With broader clinical application, DOX was found to exert varying degrees of toxicity on multiple organs, including the heart, brain, kidneys, and liver. Among these, DOX-induced cardiotoxicity is widely recognized as a major dose-limiting factor, as the heart is particularly sensitive to its toxic effects. DOX can induce cardiomyocyte hypertrophy and ultrastructural alterations, notably affecting mitochondrial integrity. The underlying mechanisms may involve enhanced redox cycling with NADH dehydrogenase, altered expression of mitochondrial proteins, DNA damage, and the activation of autophagic pathways. Regarding neurotoxicity, DOX has been shown to stimulate the production of tumor necrosis factor-alpha (TNF-α), activating microglial cells in the brain, releasing pro-inflammatory cytokines. Elevated TNF-α upregulates inducible nitric oxide synthase (iNOS), increasing reactive nitrogen species (RNS) levels and promoting nitration of manganese superoxide dismutase (MnSOD). This nitration reduces MnSOD activity, producing elevated reactive oxygen species (ROS). Excess ROS facilitates the opening of the mitochondrial permeability transition pore (PTP), triggering cytochrome c release and initiating apoptosis. Regarding nephrotoxicity, DOX can damage glomerular podocytes, contributing to renal pathologies. Clinically, this manifests as severe proteinuria, renal enlargement, and increased glomerular capillary permeability. Mitochondrial dysfunction and lipid peroxidation are also implicated in the pathogenesis of DOX-induced nephropathy. With respect to hepatotoxicity, DOX-induced ROS generation leads to elevated activities of superoxide dismutase (SOD) and glutathione peroxidase (GPX), increased DNA damage, and depletion of key antioxidants such as glutathione (GSH) and vitamin E. These effects contribute to oxidative stress and liver injury. Moreover, ROS may activate IκB kinase (IKK), which promotes the expression of pro-inflammatory cytokines and ultimately leads to hepatocyte death [92]. In monitoring adverse reactions during DOX treatment for HCC, mild vomiting and hair loss are relatively common. At the same time, cardiotoxicity occurs more frequently and is generally more severe compared to other toxicities [81,93,94].

In the clinical monitoring of adverse reactions to MTX in the treatment of HCC, typical side effects include vomiting and alopecia. However, bone marrow suppression and cardiotoxicity are also frequently observed and may be life-threatening [84,86,95]. Although the precise mechanisms underlying MTX-induced cardiotoxicity remain incompletely understood, several contributing factors have been identified. These include generating reactive ROS through interactions between MTX and iron, inhibition of topoisomerase II, and mitochondrial damage—all of which contribute to oxidative stress and cardiac dysfunction [96].

The most common toxic side effect of MMC is delayed bone marrow suppression. Additionally, diarrhea, anorexia, and hair loss are also relatively common. Hair loss, rash, and stomatitis occur occasionally [97]. Other rare but potentially fatal side effects include hemolytic uremic syndrome, interstitial pneumonia, and heart failure. In clinical studies of MMC treatment for HCC, patients were observed to experience varying degrees of bone marrow suppression, and the majority of patients developed symptoms of nausea and vomiting [88,98].

In clinical trials evaluating IDA for treating various malignancies, bone marrow suppression has been identified as the primary dose-limiting toxicity. Gastrointestinal toxicity is the second most frequently reported adverse effect and has also proven dose-limiting in specific phase I studies. In addition, IDA is associated with a broad spectrum of dermatological toxicities and exhibits some degree of cardiotoxicity. Notably, extravasation of IDA during administration can result in severe local tissue damage, including ulceration and necrosis [99]. The adverse effects and toxicities associated with the above non-plant-derived quinones are summarized in Table 6.

## 5. Investigation of the Attenuation Effects of Plant-Derived Quinone Compounds on the Toxicity of the Non-Plant-Derived Quinone Compound Dox

Due to the severe side effects and high toxicity of non-plant-derived quinone-based anti-HCC drugs, recent years have witnessed notable progress in research on plant-derived quinone compounds for treating HCC. Simultaneously, some researchers have investigated whether these plant-derived quinones can help mitigate the toxic side effects associated with non-plant-derived quinone anti-HCC drugs. Most studies have centered on the detoxifying effects of DOX. One study on emodin demonstrated its ability to restore the reduced ejection fraction (EF) and end-systolic volume (ES) in DOX-treated mouse hearts. In addition, emodin significantly lowered serum levels of CK-MB, LDH, and IL-1β, alleviated cellular disarray, and corrected disorganized myocardial fiber structures, thereby improving DOX-induced cardiac dysfunction and myocardial injury. Likewise, shikonin was shown to reduce DOX-induced elevations in MDA, 4-HNE, and NADPH oxidase activity, while increasing the suppressed levels of GSH and SOD, thereby mitigating DOX-induced oxidative stress damage [100,101]. Compared to other plant-derived quinones, baicalein exhibits a broader detoxifying effect against doxorubicin (DOX)-induced toxicity. Karabulut et al. [102,103,104] investigated the detoxifying effects of thymoquinone (TQ) on DOX-induced toxicity from three perspectives: cardiotoxicity, nephrotoxicity, and reproductive toxicity. Regarding cardiotoxicity, TQ restored the myocardial fiber tissue damaged by DOX to near-normal levels and significantly reduced the expression of molecular chaperones HSP70, HSP90, and GRP78 in the cytoplasm of rat myocardial fibers. Regarding nephrotoxicity, TQ alleviated the severity of DOX-induced kidney injury and significantly increased total antioxidant status (TAS). In the context of reproductive toxicity, TQ mitigated the reduction in testicular weight caused by DOX and markedly decreased the expression levels of Caspase-3 and HSP90 in rat reproductive cells. Compared to TQ, tanshinone I (Tan I) and tanshinone IIA (Tan IIA) display a more targeted detoxifying effect against DOX-induced cardiotoxicity. Jiang, Xu, and colleagues [105,106] conducted experimental evaluations on the cardioprotective properties of Tan I and Tan IIA. Tan I was shown to dose-dependently restore the reduced ejection fraction (EF) and end-systolic volume (ES) caused by DOX and attenuate DOX-induced cardiomyocyte apoptosis. Meanwhile, Tan IIA significantly enhanced cell viability and prevented cardiomyocyte apoptosis induced by DOX. Furthermore, Tan IIA preserved myocardial architecture and prevented fiber disarray, restoring cardiac function impaired by DOX. The protective effects of the above plant-derived quinones against DOX-induced toxicity are summarized in Table 7.

## 6. Clinical Translation of Quinonoid Compounds—Challenges and Limitations

Overall, quinone-based compounds currently face significant challenges in clinical translation and further development. Although anthraquinone derivatives can alter cellular redox states through iron-dependent lipid peroxidation, increasing ROS production, and inducing oxidative stress in tumor cells, their pharmacokinetic profiles reveal poor oral absorption. As a result, these compounds are predominantly administered via intravenous injection. Moreover, their tendency to accumulate in cardiac tissue raises concerns regarding potentially severe cardiotoxicity [66]. Naphthoquinone derivatives, represented by shikonin, can undergo redox cycling in isolated mitochondria, generating ROS. However, naphthoquinones’ generally poor water solubility limits their utility in cancer therapy. Pharmacokinetically, naphthoquinones exhibit considerable variability across different compounds. For instance, shikonin demonstrates low bioavailability. To address this, approaches such as encapsulation in nanogels have been explored to enhance its stability both in vitro and in vivo [25,107]. TQ, the reduced form of thymoquinone, a representative plant-derived benzoquinone, exhibits potent antioxidant activity through its high free radical scavenging capacity. It has shown promise in treating chronic hepatic and renal injuries. However, TQ’s high lipophilicity and limited hydrophilicity present significant formulation challenges, impeding its progression into clinical trials [108]. Tan I and Tan IIA, representative plant-derived phenanthrenequinones, have relatively low toxicity profiles. Tan IIA, in particular, contains conjugated double bonds, a dihydrofuran ring, and quinone carbonyl groups that enable it to scavenge free radicals and interrupt lipid peroxidation chain reactions, thereby exerting antioxidant effects and protecting the DNA of normal cells. Nevertheless, Tan IIA’s high lipophilicity, coupled with its hydrophilic derivatives’ rapid metabolism and short half-life, compromises its biosafety and limits its clinical applicability. Similarly, Tan I benefits from a structure conducive to free radical scavenging and modulation of intracellular antioxidant signaling pathways. Unfortunately, its pronounced lipophilicity also poses significant barriers to clinical translation, necessitating further research and development [33,63].

## 7. Conclusions and Prospects

The selected non-plant-derived quinone compounds discussed in this article are widely used chemotherapeutic agents in clinical settings. Although they exhibit consistent anti-HCC efficacy and remain integral to current treatment protocols, their associated toxic side effects can significantly compromise patients’ quality of life during therapy. Consequently, current research focuses on reducing these toxicities while maintaining or enhancing therapeutic effectiveness. In addition to exploring combination therapies that achieve synergistic antitumor effects and reduced toxicity, applying advanced drug delivery technologies—such as nanoparticle-based systems—also plays a critical role in optimizing clinical outcomes. Among the four non-plant-derived quinone compounds, DOX has garnered significant attention from researchers in recent years. Numerous studies have focused on the development of drug delivery systems based on nanoproteins or metal–organic frameworks to enhance the anti-HCC efficacy of DOX while minimizing its associated side effects. IDA, often regarded as a clinical “counterpart” to DOX, has been the subject of multiple comparative clinical studies in recent years. Notably, IDA has demonstrated increasing advantages over DOX in TACE therapy, gradually emerging as a potential substitute [91,109,110,111]. In contrast, in recent years, mitoxantrone and mitomycin have received relatively limited clinical research attention in the context of HCC treatment. Further optimization of their therapeutic efficacy and safety profiles is warranted to fully explore their potential in anti-HCC strategies. Research on plant-derived quinone compounds for the treatment of HCC has steadily increased in recent years. These studies aim to demonstrate such compounds’ inhibitory or therapeutic effects on HCC cells and liver tumors through in vitro and in vivo experiments, pathological evaluations, and biochemical marker analyses. In parallel, growing attention is being directed toward their potential to enhance the efficacy and reduce the toxicity of conventional chemotherapeutic agents. However, due to the limited depth of investigation into the anti-HCC properties of these compounds, most available studies remain restricted to cell-based and animal models, and their overall number is still relatively small. Among the six plant-derived quinone compounds, only emodin and Tan I and Tan II are potentially present in traditional Chinese medicinal formulations such as Dahuang Zhechong Formula and Danshen Injection, respectively. However, current clinical evidence does not conclusively attribute the therapeutic effects of these formulations to the presence of these specific compounds. As a result, the pharmacological profiles of plant-derived quinones remain largely underexplored, and further investigation is warranted. Nonetheless, PLB has attracted increasing academic interest in recent years, particularly in the context of HCC. A growing body of research has focused on elucidating its pharmacodynamics and underlying mechanisms of action, including novel pathways such as cuproptosis [17]. This surge in mechanistic studies suggests that PLB may hold greater developmental potential than other plant-derived quinones, positioning it as a promising candidate for future anti-HCC drug development. Moreover, the number of plant-derived quinones conclusively shown to possess anti-HCC activity remains limited. Therefore, it is essential to expand the scope of investigation into plant-derived quinone compounds to identify additional candidates with potential for anti-HCC applications. Simultaneously, more extensive and in-depth in vitro and in vivo studies are required to elucidate further the mechanisms by which plant-derived quinones with established research backgrounds exert their effects, aiming to advance these compounds toward clinical evaluation. Additionally, considering that several of the plant-derived quinones discussed in this article have demonstrated varying degrees of toxicity mitigation when used in combination with DOX—a non-plant-derived quinone with well-documented anti-HCC efficacy—future research should also explore the potential of these compounds as adjuvants to reduce associated toxicity while maintaining or enhancing their therapeutic effects. Moreover, the combination of PLB and DOX synergistically inhibited the migration and proliferation of drug-resistant HCC cells [31]. We hypothesize that combining plant-derived quinones with DOX may mitigate DOX-induced toxicity and enhance the overall anti-HCC efficacy of both agents.

Furthermore, we speculate that these plant-derived quinone compounds may also exert detoxifying effects on other non-plant-derived quinones mentioned in this study. Their combined use could potentially lead to improved therapeutic outcomes in treating HCC. In summary, quinone compounds—especially those derived from plants—still hold significant research value and development potential in HCC treatment.

## Figures and Tables

**Table 1:** The source of plant-derived quinone compounds and their animal and cell experiments in the treatment of HCC.

Medicine	Model	Administration and Relevant Experiments	Therapeutic Effect	Reference
Emodin	SD rats	Administration: Oral gavage at 40 mg/kg/day. Relevant experiments: Liver morphology and nodule formation, and histopathological and immunohistochemical analyses were examined. Oxidative stress levels and antioxidant activity were also evaluated. In addition, the expression of angiogenesis- and oxidative stress–related proteins, such as ADAMTS-4 and MMP3, was assessed using Western blotting and RT-PCR.	The treatment significantly reduced the number of hepatic nodules and improved the structural integrity of hepatocytes in HCC-bearing rats. It also downregulated the mRNA and protein expression levels of PKCδ, ERK5, ADAMTS-4, MMP3, and VEGF in the liver, suggesting a potential inhibitory effect on angiogenesis in HCC.	[19]
	Hep-G2 cells	Administration: 60 μM Relevant experiments: Transwell migration and invasion assays, Ad-mCherry-GFP-LC3B transfection assay, validation of the PI3K-AKT signaling pathway, RNA sequencing, and other cell-based experiments.	Emodin significantly inhibits the proliferation of Hep-G2 cells and reduces the migration and invasion abilities of Hep-G2 cells. Moreover, emodin regulates the expression of proteins related to EMT, autophagy, and the PI3K/AKT/mTOR signaling pathway.	[18]
Emodin + Sorafenib	nude mice	Administration: Emodin was administered via intraperitoneal injection at 10 mg/kg/day, and sorafenib at 5 mg/kg/day. Relevant experiments: Tumor size was measured using calipers, and protein expression was assessed by immunohistochemistry.	Emodin can enhance the anticancer efficacy of sorafenib by inhibiting tumor growth and inducing apoptosis in tumor cells.	[20]
	Hep-G2 cells	Administration: 20 μM Emodin, 2 μM Sorafenib Relevant experiments: The main experiments conducted included cell cycle analysis, Ki67 cell proliferation assay, Western blotting, apoptosis detection, intracellular cholesterol measurement, and qRT-PCR.	Emodin synergistically enhances the inhibitory effect of sorafenib on liver cancer cells. Moreover, the combination treatment more effectively suppresses cholesterol synthesis, induces cell cycle arrest and apoptosis, and inhibits both the STAT3 and AKT signaling pathways.	
Emodin + Cisplatin	Hep-G2 cells	Administration: Emodin at 6.25, 25, and 50 μg/mL; cisplatin at 2.5 μg/mL. Relevant experiments: The main experiments conducted included wound healing assay, Transwell assay, gelatin zymography to detect the activities of matrix metalloproteinase-2 (MMP-2) and matrix metalloproteinase-9 (MMP-9), ELISA, and immunofluorescence analysis of E-cadherin and vimentin expression.	When combined with cisplatin, emodin can inhibit the EMT process in Hep-G2 cells by downregulating the protein expression of MMP-2 and MMP-9 and upregulating E-cadherin, thereby suppressing cell migration.	[24]
Shikonin	HCCLM3 cells Hep-G2 cells	Administration: Shikonin at 0.5, 2 μM Relevant experiments: The main assessments include apoptosis detection, mitochondrial membrane potential and mass assay, cellular oxygen consumption measurement, and ATP quantification.	Shikonin can induce oxidative stress in HCCLM3 cells and downregulate the expression of Bcl-2 and p53, thereby promoting apoptosis. Additionally, it inhibits cancer cell metabolism by regulating energy metabolism.	[26,27]
PLB	nude male BALB/c mice	Administration: PLB was administered via intravenous injection at 2 mg/kg/day. Relevant experiments: Tumor weight in mice was monitored, and the expression levels of GPX4, ubiquitin, and cleaved caspase-3 were evaluated by immunohistochemistry.	PLB inhibits tumor growth by promoting GPX4 degradation and inducing apoptosis, demonstrating a good safety profile.	[29]
PLB	nude male BALB/c mice	Administration: Intraperitoneal injection of PLB (4 mg/kg/day). Relevant experiments: Observation of metastatic nodules in the lungs and liver via in vivo imaging and HE staining. Immunohistochemical analysis to assess the expression of relevant proteins in liver tissue.	PLB can inhibit the progression of liver cancer and its pulmonary metastasis. Additionally, PLB effectively suppresses the EMT in liver cancer.	[30]
	Huh-7 cells	Administration: 2 μM, 4 μM, 8μM PLB Relevant experiments: The main experiments focused on investigating the effects of PLB on TGF-β–induced EMT in Huh-7 cells and the regulatory effects of PLB on the mRNA expression of EMT-related markers.	PLB effectively inhibits EMT in HCC cells, and this therapeutic effect is concentration-dependent.	
PLB	nude male BALB/c mice	Administration: Intraperitoneal injection of PLB 2 mg/kg/day, TTM: 10 mg/kg/day. Relevant experiments: Tumor volume changes were assessed, and tumor cell distribution was observed via hematoxylin and eosin staining. Molecular analyses included protein profiling, nucleic acid analysis, copper ion concentration measurement, and quantification of oxidative stress-related markers.	PLB can significantly inhibit tumor growth and trigger the Cuproptosis mechanism in liver cancer cells, enhancing cellular oxidative stress.	[17]
	Huh-7 cells PLC cells	Administration: 6 µM PLB Relevant experiments: The main experiments included cell cycle analysis, copper ion detection assays, immunofluorescence staining, transfection experiments, luciferase reporter assays, and methylation-specific PCR (MSP).	PLB inhibits the proliferation of HCC cells in a dose-dependent manner. At the same time, TTM mitigates the PLB-induced reduction in cell viability, indicating that PLB primarily suppresses HCC cell growth through cuproptosis.	
PLB + DOX	C57BL/6 mice	Administration: Intravenous injection of DOX (12 mg/kg) and PLB (2 mg/kg). Relevant experiments: Histopathological analysis of tumor tissues, Western blot analysis, and detection of apoptotic cells.	The targeted formulation prepared by combining PLB and DOX can inhibit the growth of drug-resistant tumors.	[31]
Tan I + EADM	C57BL/6J mice	Administration: Tail vein injection of Tan I (15 mg/kg) and EADM (3 mg/kg). Relevant experiments: Biochemical analysis of mouse serum, urine, and other samples. Immunohistochemical staining of xenograft tumor sections.	The combination treatment of Tan I and EADM significantly reduces tumor volume more effectively than monotherapy and induces apoptosis to a greater extent.	[37]
	Hep-G2 cells Huh-7 cells	Administration: Tan I at (40 μM); EADM at (0.25 μM) Relevant experiments: The main experiments included cell transfection, evaluation of drug combination synergy, and apoptosis analysis.	Tan I can act synergistically with EADM to enhance the inhibition of the PI3K/AKT/HIF-1α signaling pathway and reverse HIF-1α–mediated drug resistance.	
Tan IIA	C57BL6/J mice	Administration: Oral gavage of Tan IIA (low dose: 45 mg/kg, high dose: 90 mg/kg) and RMP1-14 (5 mg/kg). Relevant experiments: Conducted fluorescence scanning of mouse abdominal tumors, tumor cell apoptosis detection (flow cytometry analysis), immunofluorescence staining, and ultrasound Doppler blood flow measurement.	Tan IIA can significantly inhibit tumor growth, improve the immune microenvironment, enhance the therapeutic efficacy of PD-1 inhibitors, and simultaneously improve tumor vascular function and promote vascular normalization.	[40]
Tan IIA + Sorafenib/SC-1	Huh-7 cells Hep-G2 cells	Administration: Tan IIA (1.5 μg/mL), sorafenib (2.5 μM; 5 μM), and SC-1 (5 μM) Relevant experiments: The main experiments included flow cytometry analysis (for apoptosis detection and sub-G1 cell population analysis), cell migration, and invasion assays.	The combination of Tan IIA with sorafenib or SC-1 significantly enhanced the induction of the mitochondrial extrinsic apoptotic pathway in HCC cells, inhibited their migration and invasion, and markedly suppressed the STAT3 signaling pathway by downregulating the expression of phosphorylated STAT3	[41]
TQ	Adult male rats	Administration: Add TQ (5 mg/kg/day) to drinking water. Relevant experiments: Performed immunohistochemical staining, qRT-PCR analysis, and other assessments.	TQ significantly downregulated the expression of the anti-apoptotic protein Bcl-2, thereby promoting apoptosis. Additionally, TQ ameliorated multiple histopathological alterations in the hepatic parenchyma induced by DEN.	[44]
TQ + Cisplatin	Wistar rats	Administration: Oral gavage of TQ (20 mg/kg) and intraperitoneal injection of cisplatin (2 mg/kg). Relevant experiments: Performed biomarker assays (ALT, AST, AFP, etc.), detection of oxidative stress-related markers, and histological analysis.	The combination therapy group demonstrated superior liver function to the disease control and monotherapy groups, as evidenced by significantly improved ALT, AST, and other liver function markers. Moreover, the hepatic structural architecture in the combination group was markedly improved.	[46]

**Table 2:** The source of plant-derived quinone compounds and their clinical experiments in the treatment of HCC.

Compound Drug	Study Group Size	Treatment Protocol	Therapeutic Effect	Reference
Da Huang Zhe Chong Formula combined with Xiao Yao Formula	The study included 60 patients with primary HCC in stages II–III who were ineligible for surgery. After obtaining approval from the hospital’s ethics committee, the patients were divided into a treatment group and a control group, each consisting of 30 patients.	After undergoing TACE, the control group received supportive treatment including glucose and electrolyte supplementation, second-generation cephalosporins for infection prevention, and routine hepatoprotective therapy. Based on this regimen, the treatment group additionally received oral administration of a combined decoction of Xiaoyao Formula and Da Huang Zhe Chong Formula.	Significantly reduces the incidence of post-TACE syndrome in primary HCC.	[47]
Da Huang Zhe Chong Pill	In this study, 90 patients who had undergone interventional therapy for liver cancer were selected as the research subjects. Using the random number table method, they were randomly assigned to the experimental and control groups, with 45 patients in each group.	The control group received hepatoprotective treatment with compound glycyrrhizin injection. On this basis, the experimental group was additionally treated with oral administration of Da Huang Zhe Chong pills at 3 g per administration, twice daily.	Significantly improves liver function after interventional liver cancer therapy while enhancing patients’ immune function.	[48]
Da Huang Soda Tablets	In this study, a total of 76 patients with advanced primary liver cancer were enrolled, including 64 cases complicated by cirrhosis following hepatitis and 8 cases who had previously undergone transarterial embolization and chemotherapy. There were 38 patients in the treatment group and 38 in the control group. No significant differences were observed between the two groups regarding gender, age, clinical condition, disease duration, or other baseline characteristics.	Both groups received hepatoprotective, supportive, and symptomatic treatment; additionally, the treatment group was administered 2 to 5 tablets of Da Huang Soda Tablets daily for a duration of two months.	It improves symptoms such as abdominal distension and fatigue and reduces the incidence of complications, including ascites, hepatic encephalopathy, and upper gastrointestinal bleeding.	[49]
Danshen Injection	A total of 40 patients with primary liver cancer who underwent Gamma Knife radiosurgery were included in this study. They were randomly divided into two groups, the experimental group and the control group, based on a random number table method. No statistically significant differences were found in the clinical data between the two groups.	From the first day of Gamma Knife treatment, patients in the experimental group were simultaneously administered 20 mL of Compound Danshen Injection diluted in 250 mL of 5% glucose injection via intravenous infusion once daily, continuing until the completion of Gamma Knife therapy. In contrast, patients in the control group did not receive this intervention during the course of Gamma Knife treatment.	The treatment effectively increased the levels of immunoglobulins in patients with primary liver cancer, stimulated the proliferation of CD3+ T cells and CD4+ T cells, and inhibited the production of CD8+ T cells. This, in turn, enhanced the overall immune function.	[50]
Danshen Injection	A total of 64 patients with HCC were included in this study, with 34 patients in the treatment group and 30 in the control group. No significant differences were observed between the two groups regarding age, gender, disease duration, or symptoms and signs. None of the patients in either group received chemotherapy. All cases were characterized by multiple nodular tumors, making catheter-based treatments unfeasible.	Both groups received 15 mL of Huachansu Injection once daily via intravenous infusion. On this basis, the treatment group was additionally administered 60 mL of Compound Danshen Injection diluted in 500 mL of 10% glucose solution via intravenous infusion once daily. The treatment course lasted for 30 days.	The treatment enhanced cellular immunity, improved clinical symptoms and signs, and prolonged the survival time of patients.	[51]

**Table 3:** Possible adverse reactions of plant-derived quinones.

Quinone Compounds	Model	Administration and Relevant Experiments/Relevant Tests	Toxicity and Adverse Reactions	Reference
Emodin	Male ICR mice Zebrafish Female ICR mice	Administration: Emodin: Oral administration at 1000 mg/kg (Male ICR mice); Oral administration at 0–40 μM (Female ICR mice); 0–2 μg/mL (Zebrafish) Relevant experiments: The study primarily included histopathological analysis, DNA microarray profiling qPCR, assessment of embryo sac developmental potential, evaluation of the in vivo effects of emodin exposure, analysis of anti-apoptotic effects, as well as observation and documentation of zebrafish embryonic development.	It causes testicular toxicity in mice, reduces the maturation of mouse oocytes and in vitro fertilization rates, leads to early embryonic developmental damage, and adversely affects the hatching of zebrafish embryos.	[53,54,55]
Shikonin	SD rats Conventional V79 cell line Human liver microsomes	Administration: Shikonin: 0.05, 0.1 mg/L (Conventional V79 cell line); intravenous injection 4 or 8 mg/kg (SD rats); 0.2–20 μM (Human liver microsomes) Relevant experiments: The study primarily involved cytotoxicity assays, micronucleus tests, CYP enzyme activity assays, and enzyme inhibition kinetics analyses.	It possesses cytotoxicity, dermal toxicity, and hepatotoxicity, with a potential inhibitory effect on cytochrome P450, which may affect metabolism and lead to drug–drug and food–drug interaction toxicities.	[56,57,58]
PLB	Wistar rats	Administration: PZPE, PZAC, PZHY: A single oral dose of 2000 mg/kg was administered for each of the three extracts (Acute toxicity study). Apart from the control group, the remaining three groups were each further divided into three subgroups. The PZPE group received oral administration of the extract at doses of 2.75 mg/kg, 5.5 mg/kg, and 11.0 mg/kg, respectively. The other two groups were treated with their respective extracts at doses 10 times those used in the corresponding PZPE subgroups. (Subacute toxicity study) Relevant experiments: The study primarily evaluated biochemical parameters such as AST and ALT, hematological indicators including hemoglobin (Hb), and conducted histopathological examinations of the liver and kidneys in each group.	Various PLB extracts led to elevated biochemical markers such as AST and urea levels in experimental animals and induced congestion in both the liver and kidneys. In addition, the petroleum ether extract (PZPE) specifically caused a reduction in hemoglobin (Hb) levels and hematocrit.	[60]
PLB	Zebrafish embryo	Administration: PLB: 0.13, 0.26, 0.40 and 0.53 μM (Acute toxicity study); 0.4 μM PLB (RT-qPCR analysis); 0.13, 0.26 μM (In situ hybridization (ISH) analysis) Relevant experiments: The study primarily included acute toxicity assessment, evaluation of neurodevelopmental impairment, gene expression analysis (targeting genes such as gsc, pax2a, and foxb1a), micro-CT imaging to assess ocular morphology, and metabolomic analysis.	PLB exposure significantly impaired brain differentiation in zebrafish embryos and induced severe developmental abnormalities, including spinal curvature, pericardial edema, tail malformations, and pronounced cyclopia.	[61]
TQ	Pregnant Wistar rats	Administration: TQ: Except for the control group, each of the other three groups was further divided into two subgroups, which received intraperitoneal injections of thymoquinone (TQ) at doses of 15 mg/kg, 35 mg/kg, and 50 mg/kg on gestational Day 11 and Day 14, respectively. Relevant experiments: This study primarily involved measuring serum α-amylase levels, histopathological examinations, and external and skeletal evaluations of fetuses.	Pregnant mice administered 35 mg/kg and 50 mg/kg of thymoquinone (TQ) on gestational Day 11 exhibited signs of toxicity, including abdominal distension and vaginal bleeding. Fat necrosis and inflammation around the pancreas were observed in the transverse mesocolon or mesentery. However, no external or skeletal abnormalities were detected in the fetuses.	[62]
Tan IIA	Zebrafish Zebrafish embryos	Administration: Both the chorionic embryo group and the dechorionated embryo group were treated with tanshinone IIA at concentrations of 1.0 μM, 5.0 μM, 10.0 μM, 20.0 μM, and 50.0 μM. Relevant experiments: Crystallographic analysis and assessments of lethal and teratogenic effects were conducted in this study.	Zebrafish embryos with developmental defects exhibited pericardial edema, spinal curvature, and tail loss.	[65]

**Table 4:** The source of non-plant-derived quinone compounds and their animal and cell experiments in the treatment of HCC.

Quinone Compounds	Model	Processing Method/Therapy	Administration and Relevant Experiments	Therapeutic Effect	Reference
DOX	Wistar rats	Apo-transferrin and nanolactoferrin particles loaded with doxorubicin	Administration: Oral administration of DOX at a dose of 4 mg/kg or protein nanoparticles loaded with an equivalent dose of DOX. Relevant experiments: Liver nodules were counted, and pathological assessments were performed using H&E staining. In addition, RT-PCR and WB analyses were conducted to evaluate the mRNA and protein expression levels of tumor-related markers associated with angiogenesis and apoptosis, including p53 and VEGFR1.	Reduction in the number of liver nodules, increased intrahepatic DOX concentration, and upregulated expression of cancer control-related proteins.	[68]
DOX	BALB/C-nu mice	DOX-loaded liposomes modified with glycyrrhetinic acid and peanut agglutinin as ligands.	Administration: Cy7, Cy7-Lips, Cy7-GA-Lips, Cy7-PNA-Lips, and Cy7-GA/PNA-Lips were administered via tail vein injection at a dose of 10 mg (For the in vivo imaging experiments); Normal saline (NS), DOX, DOX-Lips, DOX-GA-Lips, DOX-PNA-Lips, and DOX-GA/PNA-Lips were administered via intraperitoneal injection at a dose of 5 mg/kg (For the anti-tumor efficacy experiments) Relevant experiments: The study primarily involved in vivo imaging, assessment of anti-tumor efficacy, and histopathological analysis to evaluate the formulation’s therapeutic performance and biological effects.	Cy7-GA/PNA-Lips exhibited excellent targeting ability in in vivo imaging. Moreover, this formulation achieved more than twice the tumor inhibition rate compared to free DOX, while inducing significantly lower systemic toxicity.	[72]
DOX	Hep-G2 cells	Combined with C@Fe@Cu nanocomposite materials	Administration:0.04–80 µg/mL(DOX/DOX-C@Fe@Cu NC) Relevant experiments: MTT assay was performed to evaluate cytotoxicity, and flow cytometry was used for apoptosis analysis.	Exhibits greater cytotoxicity compared to the DOX-only group.	[69]
MTX	Hep-G2 cells	In combination with the autophagy inhibitor chloroquine (CQ)	Administration: MTX: 0 to 10 μM (MTX dose–response experiment) MTX: 5 μM; CQ: 20 μM (the combination treatment experiment) MTX: 5, 7.5 and, 10 μM (MTX-induced autophagy experiment) Relevant experiments: Dose–response experiments, cell-killing specificity assays, Western blot analysis, autophagic flux detection, and apoptosis assays were performed.	By inhibiting the mTOR signaling pathway, autophagy was activated, leading to specific suppression of the growth and proliferation of HCC cells.	[77]
IDA	VX2 rabbits	Preparation of biodegradable microspheres loaded with IDA (IDA-MS) in combination with TACE	Administration: IDA-MS: 0.5 mg Relevant experiments: The main experiments included angiography, PET-CT imaging, and hematoxylin and eosin (H&E) staining analysis.	Inhibits tumor growth and improves histopathological conditions.	[81]
	C57BL/6 mice	Combination of IDA-MS-TACE with anti-PD-1 immunotherapy	Administration: IDA-MS/IDA: 4 mg/kg Relevant experiments: The main experiments included periodic measurement of tumor size, histological analysis via hematoxylin and eosin (H&E) staining, immunohistochemistry, and mass cytometry analysis.	Enhances CD8+ T cell expression and improves the tumor microenvironment.	

**Table 5:** The source of non-plant-derived quinone compounds and their clinical experiments in the treatment of HCC.

Quinone Compounds and Administration Methods	Study Group Size	Treatment Protocol	Therapeutic Effect	Reference
Intravenous injection of DOX	This study included a total of 106 patients with HCC who were not candidates for surgery. Among them, 60 patients were randomly assigned to receive intravenous doxorubicin injection, while the remaining 46 patients did not receive any antitumor treatment.	In the treatment group, patients received intravenous administration of doxorubicin once every three weeks. The initial dose was 60 mg/m^2^, which was increased to 75 mg/m^2^ if well tolerated. If patients exhibited poor tolerance, the dose of doxorubicin was reduced by one-third or one-half, depending on the severity of adverse reactions. Patients in the control group did not receive any antitumor therapy and were followed up once a month, with hospitalization provided if necessary.	The median survival time was improved compared to the control group, and tumor volume was somewhat reduced. However, some patients died due to fatal complications induced by DOX.	[81]
Intravenous injection of PLD in glucose solution	As a Phase II clinical study, this research included 16 patients with HCC, who underwent 47 treatment cycles with pegylated liposomal doxorubicin.	On Day 1 of each 28-day treatment cycle, patients received an intravenous infusion of 50 mg/m^2^ pegylated liposomal doxorubicin (PLD) diluted in 250–500 mL of glucose solution.	DOX-induced side effects were alleviated.	[82]
LTLD combined with radiofrequency ablation	As part of the Phase III HEAT Study, this study enrolled a total of 701 patients. Among them, 347 patients received radiofrequency ablation (RFA) treatment, while 354 patients were treated with a combination of RFA and laparoscopic transabdominal liver decompression (LTLD).	To prevent hypersensitivity reactions, patients in the RFA + LTLD group were premedicated with dexamethasone, diphenhydramine, and chlorpheniramine, administered either orally or intravenously prior to drug infusion. In contrast, patients in the control group received placebo capsules and an intravenous infusion of either 0.9% sodium chloride solution or 5% dextrose in water (D5W). Subsequently, patients in the RFA + LTLD group received an intravenous infusion of LTLD at 50 mg/m^2^, while the control group received D5W. Fifteen minutes after the start of drug infusion, both groups underwent radiofrequency ablation (RFA).	RFA + LTLD did not demonstrate a significant benefit in the general population. However, in the subgroup of patients with a single tumor and an RFA ablation time of ≥45 min, an improvement in overall survival (OS) was observed.	[83]
Hepatic artery infusion of MTX	This study enrolled 23 patients with HCC and underwent hepatic arterial infusion of MTX every four weeks.	A catheter was percutaneously inserted into the hepatic artery via the femoral artery, followed by continuous infusion of mitoxantrone. The initial dose was 6 mg/m^2^/day for 3 consecutive days. Due to the absence of significant toxicity, the dose was subsequently increased to 10 mg/m^2^/day for 3 days. The treatment was repeated every four weeks.	The treatment demonstrated strong efficacy with relatively low toxicity.	[84]
Minimally invasive intratumoral injection of MTX	A total of 9 HCC patients who experienced treatment failure or developed severe complications were included in this study, and they were treated with mitoxantrone-based contrast agent injection therapy.	Under CT guidance, percutaneous intratumoral injection of mitoxantrone was performed. (A 50 mL intravenous contrast agent was administered, and the liver was scanned with a 10 mm collimation thickness during both the arterial and portal venous phases. The puncture site was marked based on the imaging findings. After local anesthesia, a 22-gauge needle was inserted into the center of the lesion for drug delivery).	Percutaneous injection of mitoxantrone was safe and feasible in patients with malignant liver lesions. The chemotherapeutic agent was effectively delivered to the tumor site, and biopsy confirmed tumor necrosis.	[85]
Intravenous injection of DHAD-PBCA-NPs	As a phase II clinical study, this research included 108 Chinese patients with HCC. Among them, 57 patients were assigned to the DHAD-PBCA-NPs group for treatment, while 51 were assigned to the DHAD group for treatment.	Patients in the treatment group received an intravenous injection of DHAD-PBCA-NPs at a dose of 12 mg/m^2^. The control group received an intravenous injection of free DHAD at the same dose of 12 mg/m^2^. Treatment was administered once every three weeks, and each patient received at least two treatment cycles.	Regarding disease progression and leukopenia rate, it was superior to the DHAD group.	[86]
High-dose intravenous injection of MMC	The study included 30 patients, of whom only 23 were histologically or cytologically confirmed to have HCC. Subsequently, these 23 patients received periodic intravenous infusions of MMC and follow-up visits.	Patients received MMC at 20–25 mg/m^2^ via intravenous infusion on the first day. At Week 6, toxicity was reassessed. Patients with normal blood counts and urinalysis were re-treated with MMC at 50% of the initial dose. Chemotherapy was repeated every 6 weeks at the second dose level until disease progression occurred.	It has a certain degree of tumor inhibitory effect, with toxicity and side effects milder than DOX.	[88]
Treatment with IDA microspheres combined with TACE	As a phase I, single-center, open-label, dose-escalation study, this research enrolled 21 patients with HCC. After administering a single session of transarterial chemoembolization (TACE), each patient received idarubicin-eluting bead injection therapy, with the idarubicin dosage increasing according to the modified continuous reassessment method.	Idarubicin-loaded microspheres with varying drug loads were selectively administered into each tumor-feeding artery. The microspheres were loaded with idarubicin at five doses: 5 mg, 10 mg, 15 mg, 20 mg, and 25 mg. Among the 21 patients enrolled, 9 received a dose of 5 mg, 6 received 10 mg, and 6 received 15 mg of idarubicin.	The median survival time was longer than that of conventional TACE treatment.	[90]
Combined treatment with IDA-TACE	In this study, a total of 90 patients with HCC who underwent transarterial chemoembolization (TACE) were enrolled. Among them, 30 received IDA-TACE treatment, while 60 underwent DOX-TACE treatment.	A mixture of 50 mg lyophilized doxorubicin (DOX) or 10 mg lyophilized idarubicin (IDA) with lipiodol was prepared and administered via intra-arterial injection. Embolization was then performed using gelatin sponge particles until arterial blood flow ceased for at least 10 min. Alternatively, 100μM drug-eluting beads (DEBs) loaded with DOX or IDA were infused until saturation of the tumor-feeding artery was achieved.	IDA-TACE has the potential to replace Dox-TACE.	[91]

**Table 6:** Possible adverse reactions of non-plant-derived quinones.

Quinone Compounds	Study Participants	Administration and Relevant Experiments/Relevant Tests	Toxicity and Adverse Reactions	Reference
DOX	HCC patients	Administration: Doxorubicin was administered via intravenous injection at a dose of 60 mg/m^2^. Relevant tests: Serum AFP levels, liver imaging, liver biopsy, liver function, electrocardiography (ECG), and two-dimensional echocardiography (2D Echo) tests were performed.	Nausea and vomiting, stomatitis, hair loss, gastrointestinal disturbances, cardiotoxicity, neurotoxicity, nephrotoxicity, hepatotoxicity.	[81,93,94]
MTX	HCC patients	Administration: Methotrexate (MTX) was administered via intravenous injection at a dose of 12 mg/m^2^. Relevant tests: Complete blood count (CBC), electrocardiography (ECG), and radionuclide ventriculography were performed. In addition, all adverse events occurring during the treatment process were recorded.	Vomiting, hair loss, bone marrow suppression, cardiotoxicity.	[84,86,95]
MMC	HCC patients	Administration: Nine patients received a single infusion of 20 mg of mitomycin C microcapsules, while eleven patients received a total dose ranging from 20 to 60 mg. Relevant tests: Complete blood count, urinalysis, liver function tests, electrocardiography (ECG), echocardiography, chest X-ray examination, and histological analysis were performed.	Delayed bone marrow suppression, diarrhea, anorexia, and hair loss. Occasional rash, peritonitis, rare hemolytic uremic syndrome, interstitial pneumonia, and heart failure.	[88,97,98]
IDA	HCC patients	Administration: Intravenous administration at a dose of 10–12.5 mg/m^2^ Relevant tests: Electrocardiogram (ECG) monitoring, left ventricular ejection fraction (LVEF) assessment, and blood cell count analysis were conducted. Additionally, gastrointestinal and dermatological adverse reactions, including the severity of vomiting, diarrhea, and skin ulceration, were systematically recorded.	Bone marrow suppression, gastrointestinal toxicity, skin toxicity, cardiotoxicity, tissue ulceration, and necrosis caused by extravasation and pain. Rare cases of liver failure and fatal arrhythmias.	[99]

**Table 7:** The detoxifying effect of plant-derived quinone compounds on doxorubicin.

Plant-Derived Quinone Compounds	Model	Administration and Relevant Experiments	Attenuation Effect	Reference
Emodin	Male C57BL/6 mice	Administration: DOX: 5 mg/kg (Intraperitoneal injection); Emodin: 10, 20 mg/kg (Oral gavage) Relevant experiments: Echocardiography, detection of myocardial injury biomarkers, and histopathological examinations (including HE staining, Immunohistochemical (IHC) staining, and TUNEL assay) were performed.	Alleviate DOX-induced myocardial injury and cardiac dysfunction.	[100]
Shikonin	Male C57BL/6 mice	Administration: DOX: 4 mg/kg (Intraperitoneal injection); Shikonin: 4 mg/kg (Intraperitoneal injection) Relevant experiments: Echocardiographic examination, hemodynamic monitoring, gene knockdown experiments (targeting Nrf2 expression), detection of cardiac injury biomarkers (such as CK-MB, ALT, and AST), and TUNEL staining were performed.	Shikonin can alleviate DOX-induced myocardial injury, which is associated with reduced inflammation and apoptosis.	[101]
	Primary cardiomyocytes from SD rats	Administration: DOX: 0.1 μM; Shikonin: 0.1 μM Relevant experiments: Western blotting (WB), RT-PCR analysis, measurement of reactive oxygen species (ROS) levels, and detection of inflammation- and apoptosis-related markers were primarily performed.	Nrf2 is involved in the protective effect of shikonin against doxorubicin-induced cardiotoxicity (following Nrf2 silencing, the protective effects of shikonin—such as the regulation of Bax and Bcl-2 expression and the reduction of caspase-3 activity—were all abolished). At the same time, Mst1 plays a key negative regulatory role in shikonin-mediated activation of Nrf2.	
TQ	Male Wistar rats	Administration: TQ group: TQ: 10 mg/kg/day (intraperitoneal injection) for 14 consecutive days; DOX group: DOX: 15 mg/kg (intraperitoneal injection on day 7); DOX+TQ group: Mice received both the TQ treatment regimen and the DOX administration as described above. Relevant experiments: The main experiments included histopathological examination, immunohistochemical (IHC) analysis, and TUNEL staining.	Alleviate DOX-induced myocardial and renal tissue damage and increase testicular weight.	[102,103,104]
Tan I	Male C57BL/6 mice	Administration: DOX: 5 mg/kg (Tail vein injection); Tan I: 5, 10 mg/kg (Oral gavage) Relevant experiments: The main evaluations included cardiac function monitoring, histopathological examination, serum biomarker analysis, oxidative stress assessment, transmission electron microscopy (TEM) observation, Western blotting (WB), and apoptosis detection.	It improved cardiac function, alleviated myocardial injury, reduced oxidative stress, and inhibited apoptosis, thereby protecting mitochondria from damage.	[105]
	H9C2 cells	Administration: DOX: 1 μM; Tan I: 10 μM Relevant experiments: The main experiments included oxidative stress assessment, mitochondrial membrane potential detection, mitochondrial reactive oxygen species (ROS) measurement, apoptosis analysis, immunofluorescence staining, Western blotting (WB), and Nrf2 knockdown experiments.	Tan I can attenuate oxidative stress, reduce apoptosis, and preserve mitochondrial function, partially through activation of the Nrf2 signaling pathway.	
Tan IIA	Male C57BL/6 mice	Administration: DOX: 3 mg/kg (Intraperitoneal injection); Tan IIA: 2.5, 5, 10 mg/kg (Low-, medium-, and high-dose groups; Intraperitoneal injection) Relevant experiments: The main experiments included echocardiographic assessment, hematoxylin and eosin (HE) staining, TUNEL staining, transmission electron microscopy (TEM) observation, and Western blot (WB) analysis.	Tan IIA alleviated DOX-induced heart failure and reversed structural alterations in the myocardium. Meanwhile, Tan IIA inhibited cardiomyocyte apoptosis by activating the DAXX/MEK/ERK1/2 signaling pathway through upregulating p-ERK1/2 and p-MEK expression.	[106]
	H9C2 cells	Administration: DOX: 60 μM/L; Tan IIA: 10, 20, 40 μM/L (TUNEL staining); 40 μM/L (RNA extraction) Relevant experiments: The main experiments included Hoechst 33342 and TUNEL staining for detecting apoptosis, RNA sequencing and bioinformatics analysis, qRT-PCR, and Western blotting, among others.	Tan IIA significantly reduces the level of caspase-3, downregulates the mRNA expression of MDR1 and MRP1, upregulates the expression of DAXX, and markedly decreases the percentage of apoptotic cells.	

## Data Availability

Not applicable.

## Abbreviations

Tan IIA	Tanshinone IIA
Tan I	Tanshinone I
PLB	Plumbagin
DOX	Doxorubicin
MTX	Mitoxantrone
IDA	Idarubicin
MMC	Mitomycin
PKC	Protein Kinase C
ERK5	Extracellular signal-regulated kinase 5
ADAMTS4	A Disintegrin And Metalloproteinase With Thrombospondin 4
MMP3	Matrix Metalloproteinase 3
VEGF	Vascular Endothelial Growth Factor
SREBP-2	Sterol Regulatory Element-Binding Protein 2
ROS	Reactive Oxygen Species
PKM2	Pyruvate kinase isozyme type M2
HIF1α	Hypoxia-inducible factor 1 alpha
ZO-1	Zona Occludens 1
ELTD1	EGF, latrophilin and seven transmembrane domain containing 1
JAK1	Janus Kinase 1
JAK2	Janus Kinase 2
STAT3	Signal Transducer and Activator of Transcription 3
AFP	Alpha-Fetoprotein
AFP-L3	Alpha-Fetoprotein isoform L3
GPC3	Glypican 3
Bcl-2	B-cell lymphoma-2
Bax	Bcl-2 associated X protein
TACE	Transcatheter Arterial Chemoembolization
ALT	Alanine Aminotransferase
TBIL	Total Bilirubin
DHAD	Dihydroxyanthracenedione
DHAD-loaded PBCA nanoparticles	Mitoxantrone-loaded Polybutylcyanacrylate Nanoparticles
HCC	Hepatocellular Carcinoma
TNF-α	Tumor Necrosis Factor-alpha
iNOS	Inducible Nitric Oxide Synthase
RNS	Reactive Nitrogen Species
MnSOD	Manganese Superoxide Dismutase
PTP	Permeability Transition Pore
SOD	Superoxide Dismutase
GPX	Glutathione Peroxidase
GSH	Glutathione
IKK	IκB kinase
HSP70	Heat Shock Protein 70
HSP90	Heat Shock Protein 90
TAS	Total Antioxidant Status
TQ	Thymoquinone
UGT	Uridine 5′-diphosphate Glucuronosyltransferase
MDR	Multidrug Resistance
IDA-MS	Biodegradable Microsphere Loaded with Idarubicin
PLD	Pegylated Liposomal Doxorubicin
RFA	Radiofrequency Ablation
LTLD	Lyso-thermosensitive Liposomal Doxorubicin
EF	Ejection FractionES: End-systolic Volume
CK-MB	Creatine Kinase-MB
LDH	Lactate Dehydrogenase
IL-1β	Interleukin-1 beta
GRP78	Glucose Regulated Protein 78
AKT	Oncogenic protein kinase B
EMT	Epithelial-mesenchymal transition
NHEJ	Non-homologous end joining
HR	Homologous recombination
TILs	Tumor-infiltrating lymphocytes
GA	Glycyrrhetinic acid
PNA	Peanut agglutininCQ: Chloroquine
GPX4	Glutathione peroxidase 4
DLAT	Lipoylated dihydrolipoamide S-acetyltransferase
DNMT1	DNA methyltransferase 1
Amr	Amrubicin
Epi	Epirubicin
Hepa1-6-R cells	DOX-resistant Hepa1-6 cells
BCLC	Barcelona Clinic Liver Cancer
MMP-2	Matrix metalloproteinase-2
MMP-9	Matrix metalloproteinase-9
p53	Tumor protein p53
MSP	Methylation-specific PCR
Hb	Hemoglobin
ECG	Electrocardiography
2D Echo	Two-dimensional echocardiography
LVEF	Left ventricular ejection fraction

## References

[1] Bray F., Laversanne M., Sung H., Ferlay J., Siegel R.L., Soerjomataram I., Jemal A. (2024). Global cancer statistics 2022: GLOBOCAN estimates of incidence and mortality worldwide for 36 cancers in 185 countries. CA A Cancer J. Clin..

[2] Qu L., Ma X., Fan D. (2021). Ginsenoside Rk3 Suppresses Hepatocellular Carcinoma Development through Targeting the Gut-Liver Axis. J. Agric. Food Chem..

[3] Rumgay H., Arnold M., Ferlay J., Lesi O., Cabasag C.J., Vignat J., Laversanne M., McGlynn K.A., Soerjomataram I. (2022). Global burden of primary liver cancer in 2020 and predictions to 2040. J. Hepatol..

[4] Koshy A. (2025). Evolving Global Etiology of Hepatocellular Carcinoma (HCC): Insights and Trends for 2024. J. Clin. Exp. Hepatol..

[5] Llovet J.M., Kelley R.K., Villanueva A., Singal A.G., Pikarsky E., Roayaie S., Lencioni R., Koike K., Zucman-Rossi J., Finn R.S. (2021). Hepatocellular carcinoma. Nature reviews. Dis. Primers.

[6] Dong M., Ming X., Xiang T., Feng N., Zhang M., Ye X., He Y., Zhou M., Wu Q. (2024). Recent research on the physicochemical properties and biological activities of quinones and their practical applications: A comprehensive review. Food Funct..

[7] Schieber A. (2018). Reactions of Quinones-Mechanisms, Structures, and Prospects for Food Research. J. Agric. Food Chem..

[8] Lemos T.L., Monte F.J., Santos A.K., Fonseca A.M., Santos H.S., Oliveira M.F., Costa S.M., Pessoa O.D., Braz-Filho R. (2007). Quinones from plants of northeastern Brazil: Structural diversity, chemical transformations, NMR data and biological activities. Nat. Prod. Res..

[9] Gomes de Carvalho N.K., Wellisson da Silva Mendes J., Martins da Costa J.G. (2023). Quinones: Biosynthesis, Characterization of (13) C Spectroscopical Data and Pharmacological Activities. Chem. Biodivers..

[10] Wang C., Ding X., Feng S.X., Guan Q., Zhang X.P., Du C., Di Y.T., Chen T. (2015). Seven New Tetrahydroanthraquinones from the Root of Prismatomeris connata and Their Cytotoxicity against Lung Tumor Cell Growth. Molecules.

[11] Tian W., Wang C., Li D., Hou H. (2020). Novel anthraquinone compounds as anticancer agents and their potential mechanism. Future Med. Chem..

[12] Lee C.L., Chang F.R., Yen M.H., Yu D., Liu Y.N., Bastow K.F., Morris-Natschke S.L., Wu Y.C., Lee K.H. (2009). Cytotoxic phenanthrenequinones and 9,10-dihydrophenanthrenes from *Calanthe arisanensis*. J. Nat. Prod..

[13] Kang J., Zhang P., Gao Z., Zhang J., Yan Z., Wang H., Chen R. (2016). Naphthohydroquinones, naphthoquinones, anthraquinones, and a naphthohydroquinone dimer isolated from the aerial parts of *Morinda parvifolia* and their cytotoxic effects through up-regulation of p53. Phytochemistry.

[14] Li Y., Zhu J., Yu Z., Zhai F., Li H., Jin X. (2023). Regulation of apoptosis by ubiquitination in liver cancer. Am. J. Cancer Res..

[15] Liu Y., Hao C., Li L., Zhang H., Zha W., Ma L., Chen L., Gan J. (2023). The Role of Oxidative Stress in the Development and Therapeutic Intervention of Hepatocellular Carcinoma. Curr. Cancer Drug Targets.

[16] Giannelli G., Koudelkova P., Dituri F., Mikulits W. (2016). Role of epithelial to mesenchymal transition in hepatocellular carcinoma. J. Hepatol..

[17] Wang C., Wang H., Wang C., Tian T., Jin A., Liu Y., Huo R., Liu T., Pan B., Guo W. (2025). Plumbagin Triggers Cuproptosis in Hepatocellular Carcinoma (HCC) via the DNA-Methyltransferase 1 (DNMT1)/microRNA-302a-3p (miR-302a-3p)/ATPase Copper Transporting Beta (ATP7B) Axis. MedComm.

[18] Qin B., Zeng Z., Xu J., Shangwen J., Ye Z.J., Wang S., Wu Y., Peng G., Wang Q., Gu W. (2022). Emodin inhibits invasion and migration of hepatocellular carcinoma cells via regulating autophagy-mediated degradation of snail and β-catenin. BMC Cancer.

[19] Hassan H.M., Hamdan A.M., Alattar A., Alshaman R., Bahattab O., Al-Gayyar M.M.H. (2024). Evaluating anticancer activity of emodin by enhancing antioxidant activities and affecting PKC/ADAMTS4 pathway in thioacetamide-induced hepatocellular carcinoma in rats. Redox Rep. Commun. Free Radic. Res..

[20] Kim Y.S., Lee Y.M., Oh T.I., Shin D.H., Kim G.H., Kan S.Y., Kang H., Kim J.H., Kim B.M., Yim W.J. (2018). Emodin Sensitizes Hepatocellular Carcinoma Cells to the Anti-Cancer Effect of Sorafenib through Suppression of Cholesterol Metabolism. Int. J. Mol. Sci..

[21] Gentilin E., Simoni E., Candito M., Cazzador D., Astolfi L. (2019). Cisplatin-Induced Ototoxicity: Updates on Molecular Targets. Trends Mol. Med..

[22] Giacomini I., Ragazzi E., Pasut G., Montopoli M. (2020). The Pentose Phosphate Pathway and Its Involvement in Cisplatin Resistance. Int. J. Mol. Sci..

[23] Sheng J., Shen L., Sun L., Zhang X., Cui R., Wang L. (2019). Inhibition of PI3K/mTOR increased the sensitivity of hepatocellular carcinoma cells to cisplatin via interference with mitochondrial-lysosomal crosstalk. Cell Prolif..

[24] Yang M., Xiong Z., Deng H., Chen X., Lai Q., Wang H., Leng Y. (2023). Effect of emodin combined with cisplatin on the invasion and migration of HepG2 hepatoma cells. J. Physiol. Pharmacol. Off. J. Pol. Physiol. Soc..

[25] Wang F., Yao X., Zhang Y., Tang J. (2019). Synthesis, biological function and evaluation of Shikonin in cancer therapy. Fitoterapia.

[26] Liu B., Jin J., Zhang Z., Zuo L., Jiang M., Xie C. (2019). Shikonin exerts antitumor activity by causing mitochondrial dysfunction in hepatocellular carcinoma through PKM2-AMPK-PGC1α signaling pathway. Biochem. Cell Biol..

[27] Yang W., Liu J., Hou L., Chen Q., Liu Y. (2021). Shikonin differentially regulates glucose metabolism via PKM2 and HIF1α to overcome apoptosis in a refractory HCC cell line. Life Sci..

[28] Liu H., Zhang W., Jin L., Liu S., Liang L., Wei Y. (2023). Plumbagin Exhibits Genotoxicity and Induces G2/M Cell Cycle Arrest via ROS-Mediated Oxidative Stress and Activation of ATM-p53 Signaling Pathway in Hepatocellular Cells. Int. J. Mol. Sci..

[29] Yao L., Yan D., Jiang B., Xue Q., Chen X., Huang Q., Qi L., Tang D., Chen X., Liu J. (2023). Plumbagin is a novel GPX4 protein degrader that induces apoptosis in hepatocellular carcinoma cells. Free Radic. Biol. Med..

[30] Du Y.Q., Yuan B., Ye Y.X., Zhou F.L., Liu H., Huang J.J., Wei Y.F. (2024). Plumbagin Regulates Snail to Inhibit Hepatocellular Carcinoma Epithelial-Mesenchymal Transition in vivo and in vitro. J. Hepatocell. Carcinoma.

[31] Cao C., Li Y., Shi F., Jiang S., Li Y., Yang L., Zhou X., Gao Y., Tang F., Li H. (2024). Nano co-delivery of doxorubicin and plumbagin achieves synergistic chemotherapy of hepatocellular carcinoma. Int. J. Pharm..

[32] Bai Y., Wen H., Lin J., Liu X., Yu H., Wu M., Wang L., Chen D. (2024). Tanshinone I improves renal fibrosis by promoting gluconeogenesis through upregulation of peroxisome proliferator-activated receptor-γ coactivator 1α. Ren. Fail..

[33] Huang X., Deng H., Shen Q.K., Quan Z.S. (2022). Tanshinone IIA: Pharmacology, Total Synthesis, and Progress in Structure-modifications. Curr. Med. Chem..

[34] Liu X., Liu J. (2020). Tanshinone I induces cell apoptosis by reactive oxygen species-mediated endoplasmic reticulum stress and by suppressing p53/DRAM-mediated autophagy in human hepatocellular carcinoma. Artif. Cells Nanomed. Biotechnol..

[35] Qian Z., Feng N., Geng A. (2023). Tanshinone I suppresses hepatocellular carcinoma cells growth through targeting DNA double-strand break repair. Cancer Biol. Ther..

[36] Jiang R., Zhang X., Li Y., Zhou H., Wang H., Wang F., Ma H., Cao L. (2020). Identification of the molecular mechanisms of Salvia miltiorrhiza relevant to the treatment of osteoarthritis based on network pharmacology. Discov. Med..

[37] Zhao J., Lin E., Cai C., Zhang M., Li D., Cai S., Zeng G., Yin Z., Wang B., Li P. (2022). Combined Treatment of Tanshinone I and Epirubicin Revealed Enhanced Inhibition of Hepatocellular Carcinoma by Targeting PI3K/AKT/HIF-1α. Drug Des. Dev. Ther..

[38] Santoni M., Rizzo A., Mollica V., Matrana M.R., Rosellini M., Faloppi L., Marchetti A., Battelli N., Massari F. (2022). The impact of gender on The efficacy of immune checkpoint inhibitors in cancer patients: The MOUSEION-01 study. Crit. Rev. Oncol./Hematol..

[39] Wang W.Q., Liu L., Sun H.C., Fu Y.L., Xu H.X., Chai Z.T., Zhang Q.B., Kong L.Q., Zhu X.D., Lu L. (2012). Tanshinone IIA inhibits metastasis after palliative resection of hepatocellular carcinoma and prolongs survival in part via vascular normalization. J. Hematol. Oncol..

[40] Mao D., Wang H., Guo H., Che X., Chen M., Li X., Liu Y., Huo J., Chen Y. (2024). Tanshinone IIA normalized hepatocellular carcinoma vessels and enhanced PD-1 inhibitor efficacy by inhibiting ELTD1. Phytomed. Int. J. Phytother. Phytopharm..

[41] Chiu C.M., Huang S.Y., Chang S.F., Liao K.F., Chiu S.C. (2018). Synergistic antitumor effects of tanshinone IIA and sorafenib or its derivative SC-1 in hepatocellular carcinoma cells. OncoTargets Ther..

[42] Mohammed N.K., Abd Manap M.Y., Tan C.P., Muhialdin B.J., Alhelli A.M., Meor Hussin A.S. (2016). The Effects of Different Extraction Methods on Antioxidant Properties, Chemical Composition, and Thermal Behavior of Black Seed (*Nigella sativa* L.) Oil. Evid.-Based Complement. Altern. Med. eCAM.

[43] Entok E., Ustuner M.C., Ozbayer C., Tekin N., Akyuz F., Yangi B., Kurt H., Degirmenci I., Gunes H.V. (2014). Anti-inflammatuar and anti-oxidative effects of *Nigella sativa* L.: 18FDG-PET imaging of inflammation. Mol. Biol. Rep..

[44] Ibrahim S., Fahim S.A., Tadros S.A., Badary O.A. (2022). Suppressive effects of thymoquinone on the initiation stage of diethylnitrosamine hepatocarcinogenesis in rats. J. Biochem. Mol. Toxicol..

[45] Tadros S.A., Attia Y.M., Maurice N.W., Fahim S.A., Abdelwahed F.M., Ibrahim S., Badary O.A. (2022). Thymoquinone Suppresses Angiogenesis in DEN-Induced Hepatocellular Carcinoma by Targeting miR-1-3p. Int. J. Mol. Sci..

[46] Farghaly M.E., Khowailed A.A., Aboulhoda B.E., Rashed L.A., Gaber S.S., Ashour H. (2022). Thymoquinone Potentiated the Anticancer Effect of Cisplatin on Hepatic Tumorigenesis by Modulating Tissue Oxidative Stress and Endoplasmic GRP78/CHOP Signaling. Nutr. Cancer.

[47] Hou B.S., Wang H.F., XU H.J., Tan Z.X., Zhan W.Y., Song Y.Q., Zhang Y.M., Liu X. (2018). Effect of Rhubarb Aphid Fanghe Xiaoyaofang Decoction on Postoperative TACE Syndrome of Primary Hepatocellular Carcinoma. Med. Inf..

[48] Tian D.X., Liu W. (2022). Effects of Dahuang Zhechong Pill on liver function and immune function after interventional therapy for liver cancer. Chin. J. Mod. Drug Appl..

[49] Deng Z.F., Zhao C. (2007). Clinical Observation of Rheum officinale Soda Tablets for treating advanced primary liver cancer. J. Pract. Med..

[50] Zhu Y.L., Yi F.T. (2020). The effect of γ knife the combined application of danshen injections on immune function in patients with primary liver cancer. Chin. J. Integr. Tradit. West. Med. Liver Dis..

[51] Zhang Q.H., Shao X.W. (2004). The clinical efficacy of compound Danshen injection in liver cancer patients and its impact on T cell subsets. Chin. J. Integr. Tradit. West. Med. Liver Dis..

[52] Semwal R.B., Semwal D.K., Combrinck S., Viljoen A. (2021). Emodin—A natural anthraquinone derivative with diverse pharmacological activities. Phytochemistry.

[53] Oshida K., Hirakata M., Maeda A., Miyoshi T., Miyamoto Y. (2011). Toxicological effect of emodin in mouse testicular gene expression profile. J. Appl. Toxicol. JAT.

[54] He Q., Liu K., Wang S., Hou H., Yuan Y., Wang X. (2012). Toxicity induced by emodin on zebrafish embryos. Drug Chem. Toxicol..

[55] Chang M.H., Chang S.C., Chan W.H. (2012). Injurious effects of emodin on maturation of mouse oocytes, fertilization and fetal development via apoptosis. Int. J. Mol. Sci..

[56] Cheng Y., Tang S., Chen A., Zhang Y., Liu M., Wang X. (2019). Evaluation of the inhibition risk of shikonin on human and rat UDP-glucuronosyltransferases (UGT) through the cocktail approach. Toxicol. Lett..

[57] Figat R., Zgadzaj A., Geschke S., Sieczka P., Pietrosiuk A., Sommer S., Skrzypczak A. (2021). Cytotoxicity and antigenotoxicity evaluation of acetylshikonin and shikonin. Drug Chem. Toxicol..

[58] Tang S., Chen A., Zhou X., Zeng L., Liu M., Wang X. (2017). Assessment of the inhibition risk of shikonin on cytochrome P450 via cocktail inhibition assay. Toxicol. Lett..

[59] Thakor N., Janathia B. (2022). Plumbagin: A Potential Candidate for Future Research and Development. Curr. Pharm. Biotechnol..

[60] Kumar D., Patil P.A., Roy S., Kholkute S.D., Hegde H.V., Nair V. (2015). Comparative toxicity profiles of Plumbago zeylanica L. root petroleum ether, acetone and hydroalcoholic extracts in Wistar rats. AYU.

[61] Lim Y., Kim C., Kim D., Kim M.J., Yu J.W., Song M.H., Kim Y., Son J., Lee J.H., Lee S.E. (2025). Plumbagin, a natural derivative of 1,4-naphthoquinone, induces cyclopic phenomenon via increased apoptosis and ROS generation in the early stage of zebrafish embryos. Ecotoxicol. Environ. Saf..

[62] AbuKhader M.M., Khater S.H., Al-Matubsi H.Y. (2013). Acute effects of thymoquinone on the pregnant rat and embryo-fetal development. Drug Chem. Toxicol..

[63] Ke L., Zhong C., Chen Z., Zheng Z., Li S., Chen B., Wu Q., Yao H. (2023). Tanshinone I: Pharmacological activities, molecular mechanisms against diseases and future perspectives. Phytomed. Int. J. Phytother. Phytopharm..

[64] Huang Y., Yu S.H., Zhen W.X., Cheng T., Wang D., Lin J.B., Wu Y.H., Wang Y.F., Chen Y., Shu L.P. (2021). Tanshinone I, a new EZH2 inhibitor restricts normal and malignant hematopoiesis through upregulation of MMP9 and ABCG2. Theranostics.

[65] Wang T., Wang C., Wu Q., Zheng K., Chen J., Lan Y., Qin Y., Mei W., Wang B. (2017). Evaluation of Tanshinone IIA Developmental Toxicity in Zebrafish Embryos. Molecules.

[66] Mattioli R., Ilari A., Colotti B., Mosca L., Fazi F., Colotti G. (2023). Doxorubicin and other anthracyclines in cancers: Activity, chemoresistance and its overcoming. Mol. Asp. Med..

[67] Tam K. (2013). The Roles of Doxorubicin in Hepatocellular Carcinoma. ADMET DMPK.

[68] Golla K., Bhaskar C., Ahmed F., Kondapi A.K. (2013). A target-specific oral formulation of Doxorubicin-protein nanoparticles: Efficacy and safety in hepatocellular cancer. J. Cancer.

[69] Saddik M.S., Elsayed M.M.A., Abdel-Rheem A.A., El-Mokhtar M.A., Mosa E.S., Al-Hakkani M.F., Al-Shelkamy S.A., Khames A., Daha M.A., Abdel-Aleem J.A. (2022). A Novel C@Fe@Cu Nanocomposite Loaded with Doxorubicin Tailored for the Treatment of Hepatocellular Carcinoma. Pharmaceutics.

[70] Chen L., Alrbyawi H., Poudel I., Arnold R.D., Babu R.J. (2019). Co-delivery of Doxorubicin and Ceramide in a Liposomal Formulation Enhances Cytotoxicity in Murine B16BL6 Melanoma Cell Lines. AAPS PharmSciTech.

[71] Sun W., Wang Y., Cai M., Lin L., Chen X., Cao Z., Zhu K., Shuai X. (2017). Codelivery of sorafenib and GPC3 siRNA with PEI-modified liposomes for hepatoma therapy. Biomater. Sci..

[72] Li X., Diao W., Xue H., Wu F., Wang W., Jiang B., Bai J., Lian B., Feng W., Sun T. (2020). Improved efficacy of doxorubicin delivery by a novel dual-ligand-modified liposome in hepatocellular carcinoma. Cancer Lett..

[73] Zhao R., Cheng S., Bai X., Zhang D., Fang H., Che W., Zhang W., Zhou Y., Duan W., Liang Q. (2024). Development of an efficient liposomal DOX delivery formulation for HCC therapy by targeting CK2α. Biotechnol. J..

[74] Fox E.J. (2004). Mechanism of action of mitoxantrone. Neurology.

[75] Ikeda M., Okusaka T., Sato Y., Furuse J., Mitsunaga S., Ueno H., Morizane C., Inaba Y., Kobayashi T., Arai Y. (2017). A Phase I/II trial of continuous hepatic intra-arterial infusion of 5-fluorouracil, mitoxantrone and cisplatin for advanced hepatocellular carcinoma. Jpn. J. Clin. Oncol..

[76] Mizushima N., Yoshimori T., Levine B. (2010). Methods in mammalian autophagy research. Cell.

[77] Xie B., He X., Guo G., Zhang X., Li J., Liu J., Lin Y. (2020). High-throughput screening identified mitoxantrone to induce death of hepatocellular carcinoma cells with autophagy involvement. Biochem. Biophys. Res. Commun..

[78] Arcamone F., Bernardi L., Giardino P., Patelli B., Marco A., Casazza A.M., Pratesi G., Reggiani P. (1976). Synthesis and antitumor activity of 4-demethoxydaunorubicin, 4-demethoxy-7,9-diepidaunorubicin, and their beta anomers. Cancer Treat. Rep..

[79] Goebel M. (1993). Oral idarubicin--an anthracycline derivative with unique properties. Ann. Hematol..

[80] Zheng Z., Ma M., Han X., Li X., Huang J., Zhao Y., Liu H., Kang J., Kong X., Sun G. (2023). Idarubicin-loaded biodegradable microspheres enhance sensitivity to anti-PD1 immunotherapy in transcatheter arterial chemoembolization of hepatocellular carcinoma. Acta Biomater..

[81] Lai C.L., Wu P.C., Chan G.C., Lok A.S., Lin H.J. (1988). Doxorubicin versus no antitumor therapy in inoperable hepatocellular carcinoma. A prospective randomized trial. Cancer.

[82] Valle J.W., Dangoor A., Beech J., Sherlock D.J., Lee S.M., Scarffe J.H., Swindell R., Ranson M. (2005). Treatment of inoperable hepatocellular carcinoma with pegylated liposomal doxorubicin (PLD): Results of a phase II study. Br. J. Cancer.

[83] Tak W.Y., Lin S.M., Wang Y., Zheng J., Vecchione A., Park S.Y., Chen M.H., Wong S., Xu R., Peng C.Y. (2018). Phase III HEAT Study Adding Lyso-Thermosensitive Liposomal Doxorubicin to Radiofrequency Ablation in Patients with Unresectable Hepatocellular Carcinoma Lesions. Clin. Cancer Res. Off. J. Am. Assoc. Cancer Res..

[84] Shepherd F.A., Evans W.K., Blackstein M.E., Fine S., Heathcote J., Langer B., Taylor B., Habal F., Kutas G., Pritchard K.I. (1987). Hepatic arterial infusion of mitoxantrone in the treatment of primary hepatocellular carcinoma. J. Clin. Oncol. Off. J. Am. Soc. Clin. Oncol..

[85] Farrés M.T., de Baere T., Lagrange C., Ramirez L., Rougier P., Munck J.N., Roche A. (1998). Percutaneous mitoxantrone injection for primary and secondary liver tumors: Preliminary results. Cardiovasc. Interv. Radiol..

[86] Zhou Q., Sun X., Zeng L., Liu J., Zhang Z. (2009). A randomized multicenter phase II clinical trial of mitoxantrone-loaded nanoparticles in the treatment of 108 patients with unresected hepatocellular carcinoma. Nanomed. Nanotechnol. Biol. Med..

[87] Mitomycin (2012). LiverTox: Clinical and Research Information on Drug-Induced Liver Injury.

[88] Cheirsilpa A., Leelasethakul S., Auethaveekiat V., Maoleekulpriroj S., Kangsumrit N., Thanakaravit P., Phanthumjida P. (1989). High-dose mitomycin C: Activity in hepatocellular carcinoma. Cancer Chemother. Pharmacol..

[89] Boulin M., Guiu S., Chauffert B., Aho S., Cercueil J.P., Ghiringhelli F., Krause D., Fagnoni P., Hillon P., Bedenne L. (2011). Screening of anticancer drugs for chemoembolization of hepatocellular carcinoma. Anti-Cancer Drugs.

[90] Boulin M., Hillon P., Cercueil J.P., Bonnetain F., Dabakuyo S., Minello A., Jouve J.L., Lepage C., Bardou M., Wendremaire M. (2014). Idarubicin-loaded beads for chemoembolisation of hepatocellular carcinoma: Results of the IDASPHERE phase I trial. Aliment. Pharmacol. Ther..

[91] Roth G.S., Teyssier Y., Abousalihac M., Seigneurin A., Ghelfi J., Sengel C., Decaens T. (2020). Idarubicin vs doxorubicin in transarterial chemoembolization of intermediate stage hepatocellular carcinoma. World J. Gastroenterol..

[92] Carvalho C., Santos R.X., Cardoso S., Correia S., Oliveira P.J., Santos M.S., Moreira P.I. (2009). Doxorubicin: The good, the bad and the ugly effect. Curr. Med. Chem..

[93] Sciarrino E., Simonetti R.G., Le Moli S., Pagliaro L. (1985). Adriamycin treatment for hepatocellular carcinoma. Experience with 109 patients. Cancer.

[94] Colombo M., Tommasini M.A., Del Ninno E., Rumi M.G., De Fazio C., Dioguardi M.L. (1985). Hepatocellular carcinoma in Italy: Report of a clinical trial with intravenous doxorubicin. Liver.

[95] Dunk A.A., Scott S.C., Johnson P.J., Melia W., Lok A.S., Murray-Lyon I., Williams R., Thomas H.C. (1985). Mitozantrone as single agent therapy in hepatocellular carcinoma. A phase II study. J. Hepatol..

[96] Damiani R.M., Moura D.J., Viau C.M., Caceres R.A., Henriques J.A.P., Saffi J. (2016). Pathways of cardiac toxicity: Comparison between chemotherapeutic drugs doxorubicin and mitoxantrone. Arch. Toxicol..

[97] Verweij J., Pinedo H.M. (1990). Mitomycin C: Mechanism of action, usefulness and limitations. Anti-Cancer Drugs.

[98] Ohnishi K., Tsuchiya S., Nakayama T., Hiyama Y., Iwama S., Goto N., Takashi M., Ohtsuki T., Kono K., Nakajima Y. (1984). Arterial chemoembolization of hepatocellular carcinoma with mitomycin C microcapsules. Radiology.

[99] Fields S.M., Koeller J.M. (1991). Idarubicin: A second-generation anthracycline. DICP Ann. Pharmacother..

[100] Dai S., Chen Y., Fan X., Han J., Zhong L., Zhang Y., Liu Q., Lin J., Huang W., Su L. (2023). Emodin attenuates cardiomyocyte pyroptosis in doxorubicin-induced cardiotoxicity by directly binding to GSDMD. Phytomed. Int. J. Phytother. Phytopharm..

[101] Tuo H., Li W., Zhao W., Zhao J., Li D., Jin L. (2024). Shikonin alleviates doxorubicin-induced cardiotoxicity via Mst1/Nrf2 pathway in mice. Sci. Rep..

[102] Karabulut D., Ozturk E., Kaymak E., Akin A.T., Yakan B. (2021). Thymoquinone attenuates doxorubicin-cardiotoxicity in rats. J. Biochem. Mol. Toxicol..

[103] Kaymak E., Öztürk E., Akİn A.T., Karabulut D., Yakan B. (2022). Thymoquinone alleviates doxorubicin induced acute kidney injury by decreasing endoplasmic reticulum stress, inflammation and apoptosis. Biotech. Histochem. Off. Publ. Biol. Stain Comm..

[104] Öztürk E., Kaymak E., Akin A.T., Karabulut D., Ünsal H.M., Yakan B. (2020). Thymoquinone is a protective agent that reduces the negative effects of doxorubicin in rat testis. Hum. Exp. Toxicol..

[105] Jiang Q., Chen X., Tian X., Zhang J., Xue S., Jiang Y., Liu T., Wang X., Sun Q., Hong Y. (2022). Tanshinone I inhibits doxorubicin-induced cardiotoxicity by regulating Nrf2 signaling pathway. Phytomed. Int. J. Phytother. Phytopharm..

[106] Xu L., He D., Wu Y., Shen L., Wang Y., Xu Y. (2022). Tanshinone IIA inhibits cardiomyocyte apoptosis and rescues cardiac function during doxorubicin-induced cardiotoxicity by activating the DAXX/MEK/ERK1/2 pathway. Phytomed. Int. J. Phytother. Phytopharm..

[107] Qiu H.Y., Wang P.F., Lin H.Y., Tang C.Y., Zhu H.L., Yang Y.H. (2018). Naphthoquinones: A continuing source for discovery of therapeutic antineoplastic agents. Chem. Biol. Drug Des..

[108] Malik S., Singh A., Negi P., Kapoor V.K. (2021). Thymoquinone: A small molecule from nature with high therapeutic potential. Drug Discov. Today.

[109] Liu H., Fan W., Li H., Qiao L., Liu Z., Zhu B., Guo J., Huang K., Tang Y., Wen J. (2025). Idarubicin versus Epirubicin in Transarterial Chemoembolization for Barcelona Clinic Liver Cancer Stage B Hepatocellular Carcinoma: An Open-label, Randomized, Phase IV Trial. Radiology.

[110] Zhao C., Yan H., Xiang Z., Wang H., Li M., Huang M. (2023). Idarubicin versus epirubicin in drug-eluting beads-transarterial chemoembolization for treating hepatocellular carcinoma: A real-world retrospective study. Investig. New Drugs.

[111] Choi J.W., Kim H.C., Han J., Jang M.J., Chung J.W. (2024). Transarterial Chemoembolization Using Idarubicin Versus Doxorubicin Chemoemulsion in Patients with Hepatocellular Carcinoma (IDADOX): Protocol for a Randomized, Non-inferiority, Double-Blind Trial. Cardiovasc. Interv. Radiol..

